# Experimental Determination of the Standard Enthalpy of Formation of Trimellitic Acid and Its Prediction by Supervised Learning

**DOI:** 10.1021/acs.jpca.3c05235

**Published:** 2024-03-06

**Authors:** Fausto Díaz-Sánchez, Miguel Angel García-Castro, María Patricia Amador-Ramírez, Jesús Andrés Arzola-Flores, Ximena Limón-Aguilar

**Affiliations:** † Facultad de Ingeniería Química de la Benemérita Universidad Autónoma de Puebla, 18 Sur y Av. San Claudio, C.P. 72570 Puebla Pue, Mexico; ‡ Facultad de Ciencias Químicas de la Benemérita Universidad Autónoma de Puebla, 14 Sur y Av. San Claudio, C.P. 72570 Puebla Pue, Mexico

## Abstract

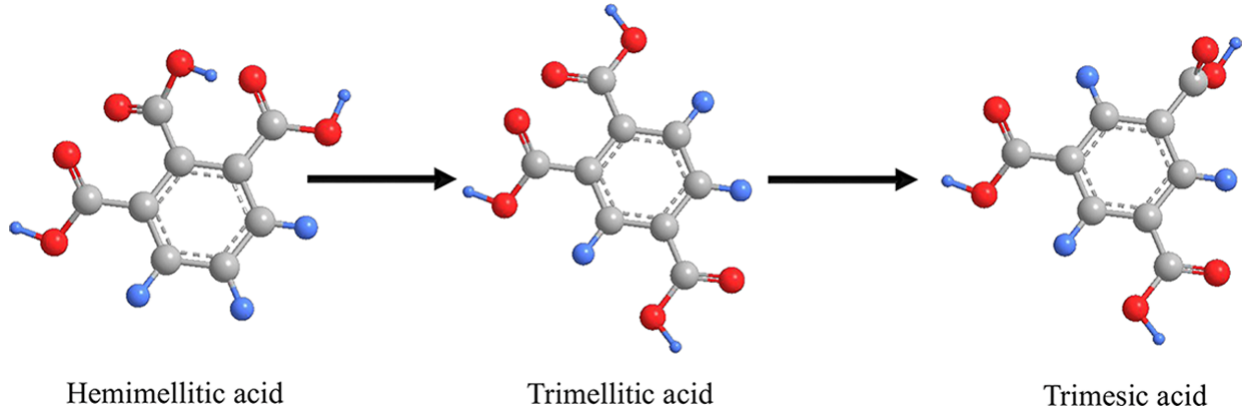

The standard molar enthalpy of formation for trimellitic acid (TMAc) in the crystalline phase at 298.15 K, Δ_f_*H*_*m*_°(cr), was calculated experimentally from the enthalpy of combustion through combustion calorimetry experiments. Likewise, the standard molar enthalpy of sublimation was determined from the standard molar enthalpy of fusion and from the standard molar enthalpy of vaporization from differential scanning calorimetry and thermogravimetry, respectively. Subsequently, the standard molar enthalpies of formation in the gas-phase at 298.15 K, Δ_f_*H*_*m*_°(g), were calculated. The enthalpies of formation for TMAc, hemimellitic, and trimesic acids were predicted using multiple linear regression (MLR) with a nonreplacement evaluation technique. MLR was applied to the data set that allowed estimating these thermochemical properties with an *R*^2^ greater than 0.99. This model was used to compare the predicted and experimental results for benzene carboxylic acids.

## Introduction

Trimellitic acid (TMAc) is a compound used as a ligand on coordination polymers,^1^ as precursors of polyester resins^2^ and polyamide-imides,^3−5^ furthermore like cross-linking agents for paints, dyes, and adhesives, and for other important chemicals production.^6,7^ In addition, the trimellitic acid particular chemical structure such as the carboxylic groups that allows the intermolecular hydrogen bonds formation renders this compound to other potential applications in organometallic and inorganic chemistry,^8^ i.e., for semisynthetic natural products,^9^ and in materials research field.^10^ A quick search for literature in SciFinder limited to the last 5 years yields more than 1400 for TMAc, which mainly corresponds to patents.

On the other hand, the chemical environment causes hydrogen bonds to have different strengths and directionalities. Consequently, TMAc derivatives along with different metals such as Cd,^11^ Zn,^12^ Al,^13^ and La^14^ are frequently used as synthetic blocking in supramolecular architectures (metal–organic frameworks; MOFs). These MOFs may present magnetic^15^ and luminescence properties,^14−16^ and their structure and topology can be controlled through the TMAs’ derivatives concentration, the solvents, and metals involved in their synthesis.^17,18^ TMAc toxicity have been subject of human health research,^19^ wherein it has been found that TMAc may cause respiratory disorders as well as allergies.

The organic compound’s thermochemical properties are usually determined experimentally by thermal and calorimetric techniques such as differential scanning calorimetry (DSC), thermogravimetry, and combustion calorimetry. The interest so far in this research is mainly to obtain enthalpies of formation in the solid and gas phases^1−5^ using computational (supervised learning)^20,21^ and functional group contribution (GC) estimation methods for experimental and a theoretical comparison.^22−24^

The supervised learning technique chosen for this work was multiple linear regression (MLR)^25,26^ to determine the groups contribution for melting, boiling points,^27−29^ enthalpies of formation,^30^ and enthalpies of combustion prediction.^31,32^ However, the functional GC models present certain limitations due to the lack of data updating, in other words, groups without values generated by the structures of recent syntheses, among others. Through supervised learning and from the MLR application,^33−35^ different parameters have been added to obtain a better acidic compounds prediction by considering the structural isomerism, as well as the previously proposed functional groups reparameterization.

In the present work, the thermochemical properties of TMAc were determined experimentally. Regarding to TMAc thermochemical properties, Yukhno G.F. and co-workers report its enthalpy of formation in the crystalline phase as −(1179.18 ± 0.79)^36^ kJ mol^–1^. In addition, in this article, the redetermination of the standard enthalpy of formation in the crystalline phase from combustion calorimetry is carried out, thus obtaining the TMAc standard enthalpy of formation in the gas phase. Also, its enthalpy of sublimation, which was obtained by thermogravimetry using the Langmuir Method, is reported.

## Methods

### Experimental Section

#### Materials and Purification

TMAc [CAS 528–44–9], from Aldrich with a mass-fraction purity of 0.99, was recrystallized three times using water, and then, it was high vacuum-dried for 2 h before being analyzed. Through this procedure, a significant increase in purity was achieved and a final value greater than 0.99 was guaranteed. Before performing the experiments, the absence of moisture for each compound was confirmed by thermogravimetry analysis (TGA) (Figure S1 in the Supporting Information); no mass loss was detected around 373 K. To confirm the compound identity, its X-ray diffractogram was obtained, as shown in Figure S2 in the Supporting Information. The purity, temperature, and enthalpy of fusion were determined by DSC using a PerkinElmer DSC 7 calorimeter and TGA (SDT) TA Instruments Q600 device (Figure S3 and Figure S4 in the Supporting Information). Each of these devices were calibrated for energy and temperature using high-purity indium. In this process, the calibration constant and the thermal resistance, necessary to correct the peak areas and the fusion temperatures, were obtained.^37^ The TMAc samples were sealed in a hermetic gold cell and subjected to heating rates of 3.0 °C min^–1^ in constant flux of a dry nitrogen atmosphere. It is convenient to note that the TMAc fusion could only be carried out in gold high pressure capsules because if aluminum hermetic capsules were used, there was a compound mass loss; thus, the capsules experimented deformation. No other thermal signal was observed during heating between 298.15 K and the compound melting temperature. The TMAc heat capacity data were determined by PerkinElmer DSC7 calorimeter. In this case, the temperature interval was from 295.15 to 411.15 at 10.0 K min^–1^ scanning rate, and a high-purity nitrogen at 30.0 cm^3^ min^–1^ flow rate was applied to the sample. Before the TMAc heat-capacity measurements were made, the DSC 7 calorimeter was tested by determining *C*_*p*_ from a sapphire high-purity sample. For this reference substance, the measured heat capacities were of 78.58 J mol^–1^ K^–1^ at 300 K, 88.71 J mol^–1^ K^–1^ at 350 K, and 95.54 J mol^–1^ K^–1^ at 400 K. Compared to the literature values of 79.43 J mol^–1^ K^–1^ at 300 K, 88.87 J mol^–1^ K^–1^ at 350 K, and 96.10 J mol^–1^ K^–1^ at 400 K,^38^ an agreement of better than 99.39% was found. In Table 1, the suppliers, purities, and methods used for obtaining the purities of all substances involved in the experiments reported in this paper are shown.

**Table 1:** Supplier, Purity, and Purification Method of Compounds

compound	CAS	supplier	initial mole fraction purity	method of purification	final mole Fraction purity^a^	method for obtain purity or composition
TMAc	528–44–9	Sigma-Aldrich	0.99	recrystallized	0.9938 ± 0.0016	DSC
paraffin oil	8012–95–1	Sigma-Aldrich				elemental analysis
indium	7440–74–6	NIST	0.999999			
aluminum oxide	1344–28–1	NIST	0.9995			
benzoic acid	65–85–0	NIST	0.999996			
phenanthrene	85–01–8	Sigma-Aldrich	0.99	sublimation	0.9997 ± 0.0001	DSC
pyrene	129–00–0	Fluka	0.99	sublimation	0.9996 ± 0.0003	DSC
anthracene	120–12–7	Sigma-Aldrich	0.99	sublimation	0.9998 ± 0.0005	DSC

a Experimental value obtained in this work. The experiments were realized to average atmospheric pressure of 78.8 kPa, *u*(*P*) = ± 1 kPa. The uncertainties correspond twice the overall standard deviation of the mean and include the contributions from the calibration and *u*(*T*) = 0.1 K.

#### Combustion Calorimetry

Due to the compound’s hygroscopic nature, the samples were subjected to a vacuum pressure before each combustion. After that, the absence of moisture was confirmed by TGA (Figure S1 in the Supporting Information). Meanwhile, the energies of combustion were obtained using an isoperibolic static bomb calorimeter. The procedure and apparatus used in this research were those described in a previous work.^39^ The calorimeter energy equivalent, ε(calor), was determined considering (10135.1 ± 2.5) J K^–1^ from seven combustion runs using NIST 39j benzoic acid. Under the conditions specified by the NIST certificate, this reference material possessed a mass energy of combustion Δ_c_*u* = −(26434.0 ± 3.0) J g^–1^ (where the uncertainty corresponds to the expanded uncertainty). The uncertainty associated with the equivalent in energy was calculated as twice the standard deviation of the mean. The seven calibration runs were carried out high purity gaseous oxygen atmosphere (Air Liquide Corp., mass fraction of 0.99999) at a pressure 3.04 MPa pressure and 1.00 cm^3^ of water added to the pump; these same conditions were used to oxidize the compounds under study. The empirical formula of our cotton-thread fuse was C_1.000_H_1.742_O_0.921_, with a specific energy of combustion Δ_c_*u*° = −(16945.2 ± 4.2) J g^–1^, where the uncertainty is the standard deviation of the mean. Paraffin oil was used as an auxiliary material to achieve the compounds’ complete oxidation. Its specific combustion energy is Δ_c_*u*° = −(46238.5 ± 6.6) J g^–1^, where the uncertainty is the standard deviation of the mean.^39^ Energy corrections were performed using the value of −59.7 kJ mol^–1^ for aqueous nitric acid formation.^40^ The samples apparent masses and materials participating in the combustion processes were measured by ME215S Sartorius balance (accuracy: ±0.01 mg). Washburn corrections were done for each substance using the values given in Table 2 to reduce the resulting energy values to standard conditions at 298.15 K. Such corrections were done according to the procedure of Hubbard and colleagues.^41^ Albeit to calculate the energy change associated with the pressure change, an estimated value of (δ*u/δp*)_T_ = −0.2 J g^–1^ MPa^–1^ at 298.15 K was used, which is a typical value for most solid organic compounds.^42^ In addition, the elements atomic weights were those recommended by IUPAC in 2021.^43^

**Table 2:** Physical Properties of the Compounds Involved in the Combustion Experiments

compound	*M*^a^ (g mol^–1^)	final mole fraction purity^b^	(δ*u*/δ*p*)_T_ (J g^–1^ MPa^–1^)	Δ_cr_^l^*H*^b^ (kJ mol^–1^)	*T*_fus_^b^ (K)	*C*_*p*_ (J g^–1^ K^–1^)	ρ (g cm^–3^)
TMAc (cr)	210.140	0.9938 ± 0.0016	–0.2	56.6 ± 1.6	507.0 ± 0.4^c^	1.18 ± 0.06^b^	1.580^e^
					521.8 ± 1.2^d^		
paraffin oil (l)	14.027		–0.257^f^			2.22^f^	0.857^f^
cotton (s)	28.502		–0.289^f^			1.67^f^	1.500^f^

a Molecular masses are based on the 2021 IUPAC recommendations.^43^

b Experimental value obtained in this work. The experiments were performed at an average atmospheric pressure of 78.8 kPa, *U*(*P*) = ± 1 kPa. The uncertainties correspond twice the overall standard deviation of the mean, and include the contributions from the calibration and *U*(*T*) = 0.1 K.

c Value obtained by the thermogram of SDT.

d Value obtained by the thermogram of DSC using high-pressure gold capsules.

e Value obtained of ref (68).

f Estimated values as in ref (69).

#### Thermogravimetric Analysis

The enthalpy of vaporization was measured by using a simultaneous DSC and TGA (SDT) TA Instruments Q600 device. The system was calibrated for mass, temperature, and heat flow as described elsewhere.^44^ The vapor pressure as a function of the temperature can be calculated from the Langmuir equation
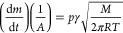
1where d*m*/d*t* is the sample mass loss rate at temperature *T*; *A* is the exposed area of vaporization and sublimation, which was here considered equivalent to the transversal section of the cylindrical alumina cup as it is calculated from its internal diameter (5.25 mm); *p* is the compound vapor pressure; *M* is the molar mass; *R* is the gas constant; and γ is the vaporization constant. By combining the corrected Clausius–Clapeyron and Langmuir expression, considering the compound diffusion effect in the gas phase, eq 2 is obtained. Where the constant *B* includes such an effect as proposed by Pieterse and Focke.^45^
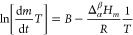
2

Finally, the substance enthalpy of vaporization or sublimation can be determined using this equation (see eq 2) and the experimental mass loss rate data at different temperatures are obtained by the SDT device. This methodology requires a device able to operate with reasonable control in a wide range of temperatures; in addition, a high sensitivity in recording mass changes is needed. The thermogravimetric system measuring reliability like the enthalpies of vaporization and sublimation was performed using three standards: pyrene, phenanthrene, and anthracene. These standard materials were purchased from Sigma-Aldrich and Fluka, respectively, were purified prior its use by repeated sublimation under vacuum at a residual pressure at 20 Pa. The obtained molar enthalpy of vaporization values at 298.15 K for pyrene were as follows: (91.4 ± 1.0) and (81.2 ± 1.4) kJ mol^–1^ for phenanthrene; meanwhile, the molar enthalpy of sublimation values at 298.15 K were as follows: (101.1 ± 5.2) and (100.2 ± 4.2) kJ mol^–1^ for pyrene and anthracene, respectively. The uncertainties were calculated from the standard deviation of the slope, which is the standard uncertainty. The values shown in Tables S1–S6 (in the Supporting Information) are in excellent agreement with literature values.^38^

### Computational Details Using Supervised Learning

#### Multiple Linear Regression

This regression model is characterized by the inclusion of multiple regressor variables;^46−48^ in other words, the dependent variable is not affected only by one independent variable, which is expressed in eq 3.
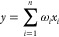
3The model relates a dependent variable (*y*) with *n* regressor variables (*x*_*i*_) and finally a weight associated with each regressor variable (ω_*i*_).^49,50^ Yielding the MLR cost function being ascribed as it is showed in eq 4.
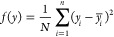
4Equation 4 shows the function that needs to be optimized when a regression-type problem is faced by the use of supervised learning; the closer this expression is to zero, the better the resulting model predictive power. In this expression, *N* represents the model data amount used, *y*_*i*_ refers to the problem actual values, and  are the values predicted by the model; thus,  can be replaced by considering eq 3, which would results on the next expression.
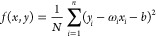
5

An important point to consider in this regression is multicollinearity, this is described as a strong correlation between the model regressor variables, which means lack of orthogonality between them,^51^ it is a data problem issue that may cause less reliability on the model parameter estimates^52^ resulting on the coefficients standard error increase.^51^ It occurs when *k* vectors lie in a subspace of dimension less than *k*,^53^ it is important that must be avoided the confusion between multicollinearity and correlation, the latter is the linear relationship between only two variables, so correlation is a special case of multicollinearity. Increasing the standard errors of the regressor variables means that some variables are statistically insignificant when in fact they should be significant in the fitting process.^54^

To avoid multicollinearity, some considerations can be applied, such as (a) increasing the sample size, (b) by suppressing variables that are correlated with others (dummy variables), (c) working with logarithmized series and, and finally (d) using cross-sectional data.^55^

Additionally, the last point to consider is the overfitting,^55^ i.e., an overfitted model produces overly optimistic data results that appear within the model, and therefore, cannot be replicated when using new future data.^56^

## Results and Discussion

### Experimental Results

Table 2 shows the purity and fusion properties for each compound. For TMAc, as explained above, the compound sublimes before it melts and vaporizes after finishing the fusion process. This causes pressure rise inside the hermetic aluminum capsule, which renders capsule deformation, and the compound mass loss, as well as an extensive melting peak signal. As a result to avoid this, a high-pressure gold capsule and a relatively high compound load (about 6 mg in four independent experiments) were used. This allowed us to obtain well-defined fusion peaks (Figure S3 in the Supporting Information); thus, the fusion enthalpy could be obtained, as revealed by the compound high purity. However, the melting temperature was shifted to a higher value, *T*_fus_ = (521.8 ± 1.2) K, compared to those reported in the literature.^57−66^ Probably, a high pressure inside the capsule was generated, which provoked the melting temperature shift. On other hand, the experiments in SDT show that TMAc starts sublimating approximately at 485.15 K, and melted at 507.0 K, consequently immediately vaporizes. Fusion temperature result was obtained from “onset point” of “heat flow” signal, as can be observed in the thermogram of Figure S4 in the Supporting Information. The average temperature of fusion, obtained from four experiments by SDT, was (507.0 ± 0.4) K. Therefore, in this work, we report two values, one obtained by DSC and the other from SDT (Table 2). Likewise, it was considered that the fusion enthalpy is not a pressure function. This assumption is based on studies of indium melting at different pressures, wherein it was found that significant changes on this parameter may provoke small shift on the material fusion enthalpy.^67^ For TMAc, the uncertainties correspond to twice the standard deviation of the mean and were obtained from independent experiments.

From the data collected by DSC, the equations of molar heat capacities as temperature functions were obtained. Equation 6 corresponds to TMAc from 295.15 to 411.15 K range (the values of *C*_*p*,*m*_ at different temperatures are shown in Table S7 in the Supporting Information).
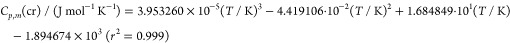
6

For the energy of combustion Δ_c_*u*° (298.15 K), all the experiments and the uncertainties assigned to twice the standard deviation of the mean, ⟨Δ_c_*u*°⟩, are shown in Table 3. In addition the ideal combustion reaction for TMAc from which the values of Δ_c_*u*° was derived are described by eq 7.
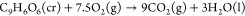
7

**Table 3:** Combustion Experiments for TMAc at 298.15 K and *p*° = 0.1 MPa^a^

	exp. 1	exp. 2	exp. 3	exp. 4	exp. 5	exp. 6	exp. 7
*m* (TMAc)/g	0.98431	0.99150	0.99156	0.99292	0.96352	0.98969	0.98211
*m* (p. oil)/g	0.15349	0.18881	0.17461	0.1923	0.1512	0.15585	0.17298
*m* (cotton)/g	0.00178	0.00170	0.00166	0.00164	0.00168	0.00179	0.00182
(platinum)/g	11.51597	11.52378	11.51775	11.5194	11.52056	11.52631	11.52174
*T*_i_/K	295.3007	295.2940	295.2910	295.3190	295.2876	295.2946	295.2943
*T*_f_/K	297.5356	297.7015	297.6321	297.7554	297.4814	297.5493	297.6154
Δ*T*_corr_/K	0.0432	0.0430	0.0406	0.0546	0.0427	0.0426	0.0423
Δ*T*_c_/K	2.1917	2.36451	2.3005	2.3818	2.1511	2.2122	2.2788
ε^i^(cont)/kJ K^–1^	0.0174	0.0175	0.0175	0.0175	0.0174	0.0174	0.0175
ε^f^(cont)/kJ K^–1^	0.0184	0.0186	0.0185	0.0186	0.0184	0.0184	0.0185
ε(calor) (Δ*T*_c_)/kJ	22.2129	23.9646	23.3158	24.1394	21.8016	22.4204	23.0956
ε(cont) (Δ*T*_c_)/kJ	0.0375	0.0408	0.0397	0.0412	0.0367	0.0378	0.0393
Δ*U*_ign_/kJ	0.0042	0.0042	0.0042	0.0042	0.0042	0.0042	0.0042
(−Δ*U*_IBP_)/kJ	22.2462	24.0012	23.3513	24.1764	21.8341	22.454	23.1307
Δ*U*(HNO_3_)/kJ	–0.0013	–0.0013	–0.0013	–0.0015	–0.0010	–0.0013	–0.0011
Δ*U*_corr_/kJ	0.0209	0.0217	0.0214	0.0218	0.0204	0.0211	0.0212
(−*m*Δ_c_*u*°) (p. oil)/kJ	7.0973	8.7304	8.0738	8.8918	6.9914	7.2064	7.9985
(−*m*Δ_c_*u*°) (cotton)/kJ	0.0305	0.0291	0.0284	0.0281	0.0288	0.0307	0.0312
(−*m*Δ_c_*u*°) (TMAc)/kJ	15.0962	15.2187	15.2264	15.2332	14.7925	15.1945	15.0787
Δ_c_*u*° (TMAc)/kJ g^–1^	–15.3368	–15.3492	–15.3560	–15.3418	–15.3526	–15.3528	–15.3534
average value <Δ_c_*u*°(298.15 K)/kJ g^–1^> –15.3489 ± 0.0054							

a *m* (TMAc) is the compound mass burnt in the experiment; *m* (cotton) is the mass of cotton burnt in the experiment; m(p. oil) is the mass of paraffin oil used in the experiment, the masses were corrected for buoyancy using densities listed in Table 2; Δ*T*c is the temperature increment corrected for adiabatic conditions; *T*_i_ and *T*_f_ are the initial and final temperatures, respectively, Δ*T*_corr_ is a correction term; ε^i^(cont) and ε^f^(cont) are the initial and final content energies. ε^i^(cont) is the equivalent initial energy, meanwhile ε^f^(cont) is its final energy both are related to the bomb; ε(calor) represents the entire system energy equivalent, less the bomb contents which is defined as ε(cont) where ε(cont)·(Δ*T*_c_) = ε^i^(cont)·(298.15 K – *T*_i_) + ε^f^(cont)·(*T*_f_ – 298.15 K – Δ*T*_corr_). Δ*U*_ign_ is the experimental energy of ignition, Δ*U*_IBP_ is the energy change for the isothermal bomb process, calculated as (−Δ*U*_IBP_) = ε(cont)·(Δ*T*_c_) + ε(calor)·(Δ*T*_c_) – Δ*U*_ign_. Δ*U*(HNO_3_) is the experimental energy of formation of nitric acid, Δ*U*_corr_ is the correction to standard states, and Δ_c_*u*° is the standard massic energy of combustion, calculated as Δ_c_*u*°(TMAc) = [Δ*U*_IBP_ – Δ*U*(HNO_3_) + Δ*U*_corr_ + (−*m*Δ_c_*u*°)(p. oil) + (−*m*Δ_c_*u*°)(cotton)]/*m*(TMAc).

In Table 4, the standard molar energy and enthalpy of combustion are listed [Δ_c_*U*_*m*_°(cr) and Δ_c_*H*_*m*_°(cr)] as well as the standard molar enthalpy of formation in the crystalline phase, Δ_f_*H*_*m*_°(cr), at 298.15 K. The uncertainty assigned for each case corresponds to the expanded standard deviation which includes calibration uncertainties and energies of the auxiliaries values used.^70−72^ To calculate Δ_f_*H*_*m*_°(cr) from Δ_c_*H*_*m*_°(cr), the standard molar enthalpies of formation of H_2_O(l) and CO_2_(g), at 298.15 K, were fixed at −(285.83 ± 0.04) and −(393.51 ± 0.13) kJ mol^–1^, respectively, the uncertainty is the sum from the expanded at 95% level including the standard enthalpy of formation of H_2_O(l) and CO_2_(g) uncertainties.^73^

**Table 4:** Derived Standard Molar Value (*p*° = 0.1 MPa) in the Crystalline Phase at 298.15 K

compound	Δ_c_*U*_*m*_° (kJ mol^–1^)	Δ_c_*H*_*m*_° (kJ mol^–1^)	Δ_f_*H*_*m*_° (kJ mol^–1^)
TMAc	–3225.4 ± 2.7	–3221.7 ± 2.7	–1177.4 ± 2.9

Regarding the standard molar enthalpy of formation in the crystalline phase, the result reported here differs from that of Yukhno and Bikkulov by 1.8 kJ mol^–1^;^36^ however, this difference is contained within the reported uncertainty. Data derived by thermogravimetry for TMAc are shown in Tables 5, S8, and S9 and Figures S6 and S7, respectively.

**Table 5:** Comparison of Standard Molar Enthalpies of Sublimation at ***T*** = 298.15 K^a^

compound	Δ_cr_^l^*H*_*m*_^o^ (***T***)^b^ (kJ mol^–1^)	Δ_l_^g^*H*_*m*_^o^ (***T***)^c^ (kJ mol^–1^)	Δ_cr_^l^H_m_^o^ (*T*) + Δ_l_^g^H_m_^o^ (*T*)	Δ_cr_^g^*H*_*m*_^o^ (***T***)^d^ (kJ mol^–1^)
		vaporization process		sublimation process
TMAc	45.4 ± 2.0	97.1 ± 1.4	142.5 ± 2.4	144.8 ± 5.4

a All uncertainties correspond to twice the combined standard.

b The standard molar enthalpy of fusion was obtained by eqs 8–10.

c The standard molar enthalpy of vaporization was obtained by eqs 11–13.

d The standard molar enthalpy of sublimation was obtained by eq 15.

The standard molar enthalpy of vaporization at mean temperature, Δ_l_^g^*H*_*m*_(*T*_*m*_), comput by eq 2 using the least-squares method, is the weighted averages from the set of all the results of each experiment, Table S8 and Figure S6 in the Supporting Information.^74^ To derive Δ_cr_^g^*H*_*m*_^o^ from Δ_l_^g^*H*_*m*_^o^ at 298.15 K for TMAc, the following equations were used
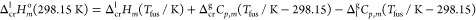
8
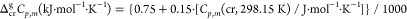
9
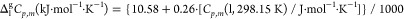
10
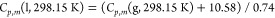
11
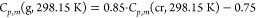
12
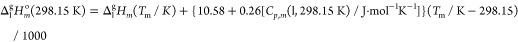
13
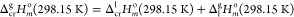
14where *T*_m_ is the vaporization mean experimental temperature. Chickos proposed eqs 8–14.^75^ The TMAc melting point value used in eq 14 was *T*_fus_ = (521.8 ± 1.2) K, which is the value obtained in the studies by DSC.

Another route followed to determine the standard sublimation enthalpy was the loss of mass before the melting temperature. The data are shown in Table S9 and Figure S7, respectively. For this acquisition the eq 15 proposed by Chickos,^76^ was used. Where *T*_av_ is the average experimental temperature of the sublimation.
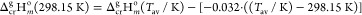
15

The standard molar enthalpies of sublimation and formation in the crystalline and gaseous phases at 298.15 K are summarized in Table 6. The assigned uncertainty is for the expanded and calorimetric energy equivalent uncertainties with a 95% level of confidence.

**Table 6:** Enthalpy of Sublimation and Standard Molar Enthalpies of Formation at 298.15 K

compound	Δ_f_*H*_*m*_°(cr) (kJ mol^–1^)	Δ_cr_^g^*H*_*m*_ (kJ mol^–1^)	Δ_f_*H*_*m*_°(g) (kJ mol^–1^)
TMAc	–1177.4 ± 2.9	144.8 ± 5.4	–1032.6 ± 6.1^a^
		142.5 ± 2.4	–1034.9 ± 3.8^b^

a Value obtained from the analysis of the sublimation process.

b Value obtained from the analysis of the vaporization process.

### Theoretical Results

By applying the Benson^76,77^ and Gani^78,79^ functional GC estimation methods, the enthalpy of formation of the TMAc was calculated in both gas and crystalline phases, in Benson’s method, GAVs taken from literature^80^ were used for COOH–COOH ortho and COOH–COOH meta corrections at the gas phase. The results obtained using these methods are shown in Tables 7 and 8.

**Table 7:** Comparison between Experimental and Estimated Values of −Δ_f_*H*_*m*_°(cr, 298.15 K) in kJ mol^–1^ by the Benson Method

compound	experimental	Benson	Δ
phthalic acid	781.2^a^	777.7	–3.5
isophthalic acid	805.3^a^	798.7	–6.6
terephthalic acid	813.6^a^	811.9	–1.7
benzoic acid	385.2^b^	386.4	1.2
2-methylbenzoic acid	416.5^b^	420.7	4.2
3-methylbenzoic acid	426.1^b^	423.7	–2.4
4-methylbenzoic acid	429.2^b^	425.7	–3.5
TMAc	1177.4^c^	1190.1	12.8

a Taken on ref (81).

b Taken on ref (76).

c Experimental value of this work.

**Table 8:** Comparison between Experimental and Estimated Values for −Δ_f_*H*_*m*_° (g, 298.15 K) in kJ mol^–1^ by Benson and Gani Methods

compound	experimental	Benson	Δ	Gani	Δ
phthalic acid	652.2^a^	643.2	–9.0	665.4	13.2
isophthalic acid	667.4^b^	669.8	2.4	667.3	–0.1
terephthalic acid	668.0^b^	672.4	4.4	667.4	–0.6
benzoic acid	295.7^c^	294.8	–0.9	294.2	–1.5
2-methylbenzoic acid	320.6^c^	325.9	5.3	324.0	3.4
3-methylbenzoic acid	327.9^c^	327.8	–0.1	325.9	–2
4-methylbenzoic acid	330.4^c^	327.2	–3.2	326.0	–4.4
TMAc	1032.6^d^	1018.2	14.4	1038.5	–5.9
	1034.9^d^		16.7		3.6

a Taken on ref (81).

b Taken on ref (80).

c Taken on ref (82).

d Experimental value of this work.

The estimation methods disadvantage is that not always all the functional group values are available, or it is necessary to recalculate for specific group studies; although a good first approximation is obtained, which is not the best, for example, from the Benson method, the estimation of a larger molecule such as TMAc results in high errors; to correct and diminish this for compounds of the family of carboxylic acids, supervised learning is applied. Table 7 shows how Gani’s method provides a good approximation with respect to the experimental data for the TMAc gas-phase enthalpy of formation; thus, the result obtained experimentally can be considered “correct”. This value obtained for TMAc is used in the training phase during the MLR application to perform the prediction of its structural isomers; meanwhile, for the TMAc crystalline phase, the experimental values of its structural isomers are already reported. Therefore, to predict the experimental value, it is necessary to be fitted to the isomers and contrast how close they are to the real value.

Using a GC type model based on the functional groups proposed by Benson^76^ and including new correction parameters, a data set was created containing 55 and 67 enthalpy of formation values at 298.15 K for the gas phase and crystalline phase, respectively, the total data set was divided into a training and testing subsets using the Hold out (70/30) evaluation technique. Based on these results, the gas and crystalline phase enthalpies of the formation of TMAc were predicted. Additionally to this, the 1,2,3-tricarboxylic benzoic acid (hemimellitic acid) and 1,3,5-tricarboxylic benzoic acid (trimesic acid) isomers prediction for the gas phase was performed since they are not reported experimentally in the literature. On the other hand, for the crystalline phase prediction, the TMAc isomers are already reported,^36^ hemimellitic acid was used to train the model, and the algorithm veracity was checked from trimesic acid, allowing the TMAc prediction to be performed.

**Figure 1:**
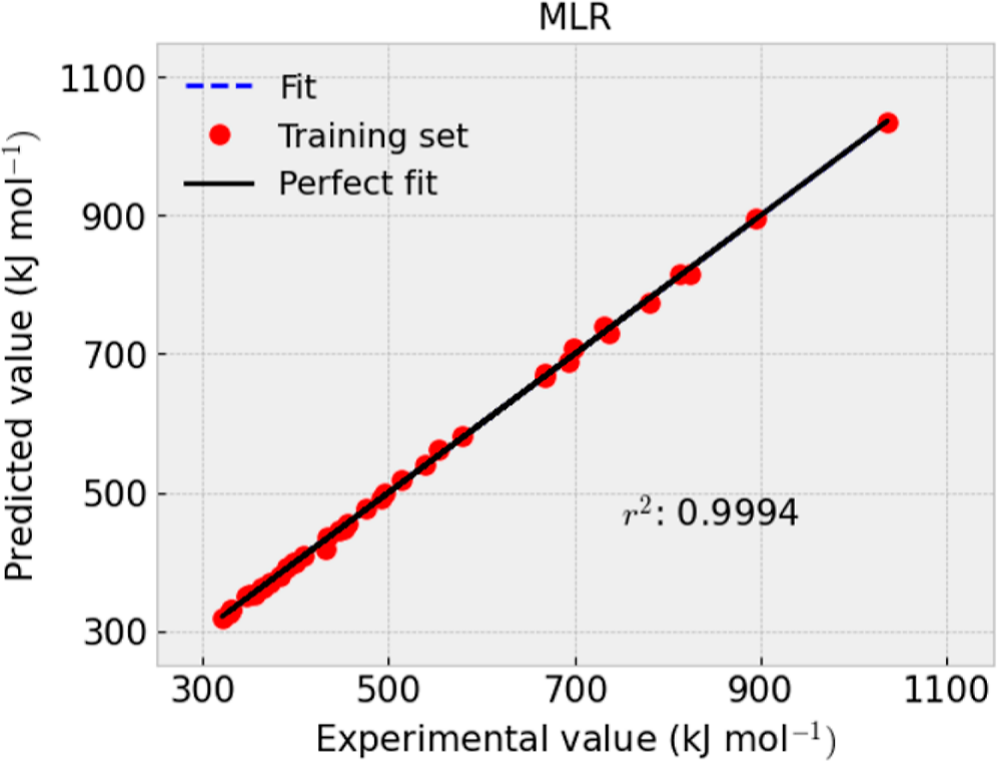
Comparison of experimental and predicted value of −Δ_f_*H*_*m*_° (g, 298.15 K) from the training set using MLR.

**Figure 2:**
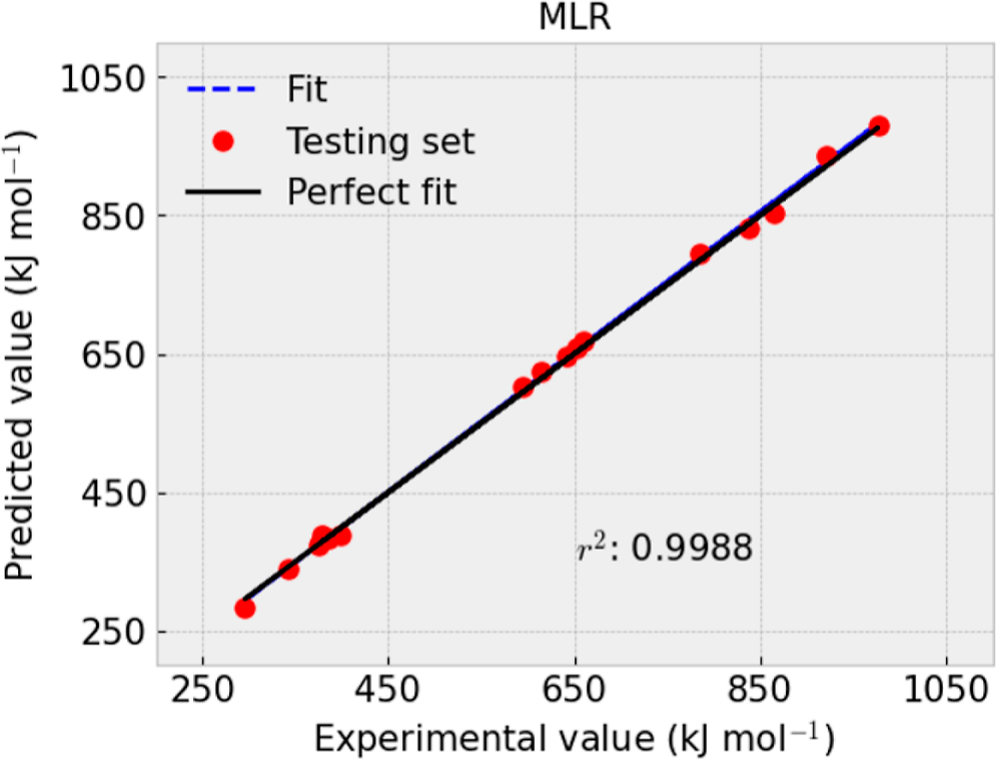
Experimental and predicted value comparison for −Δ_f_*H*_*m*_° (g, 298.15 K) with the testing set using MLR.

**Figure 3:**
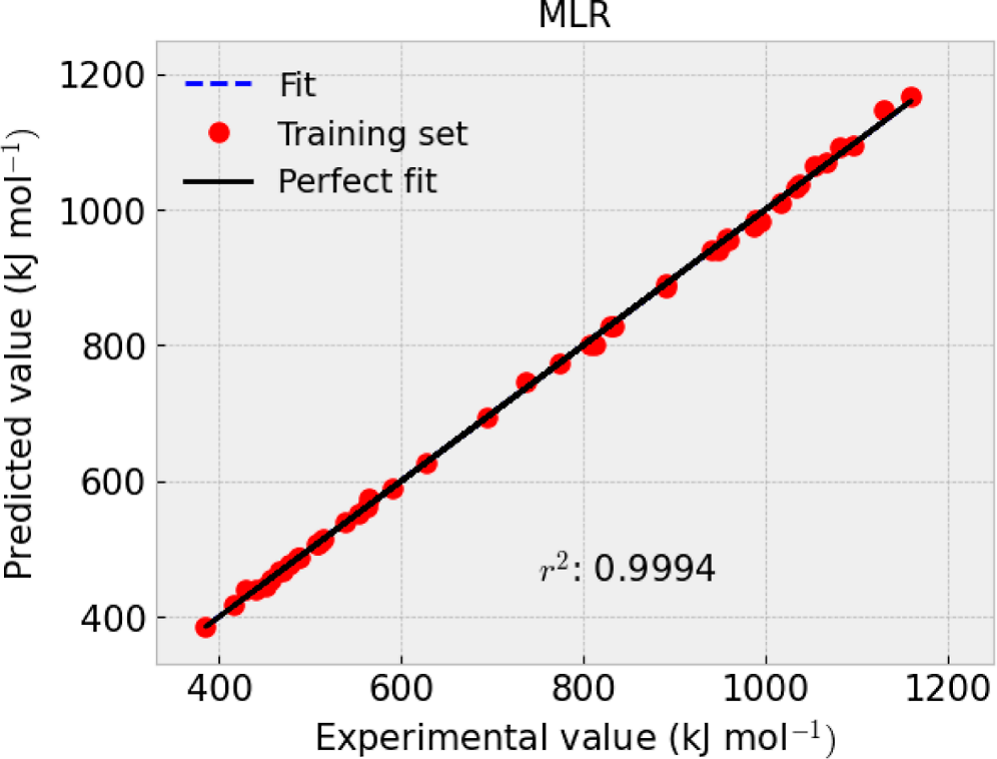
Experimental and predicted value comparison of −Δ_f_*H*_*m*_°(cr, 298.15 K) from the training set using MLR.

**Figure 4:**
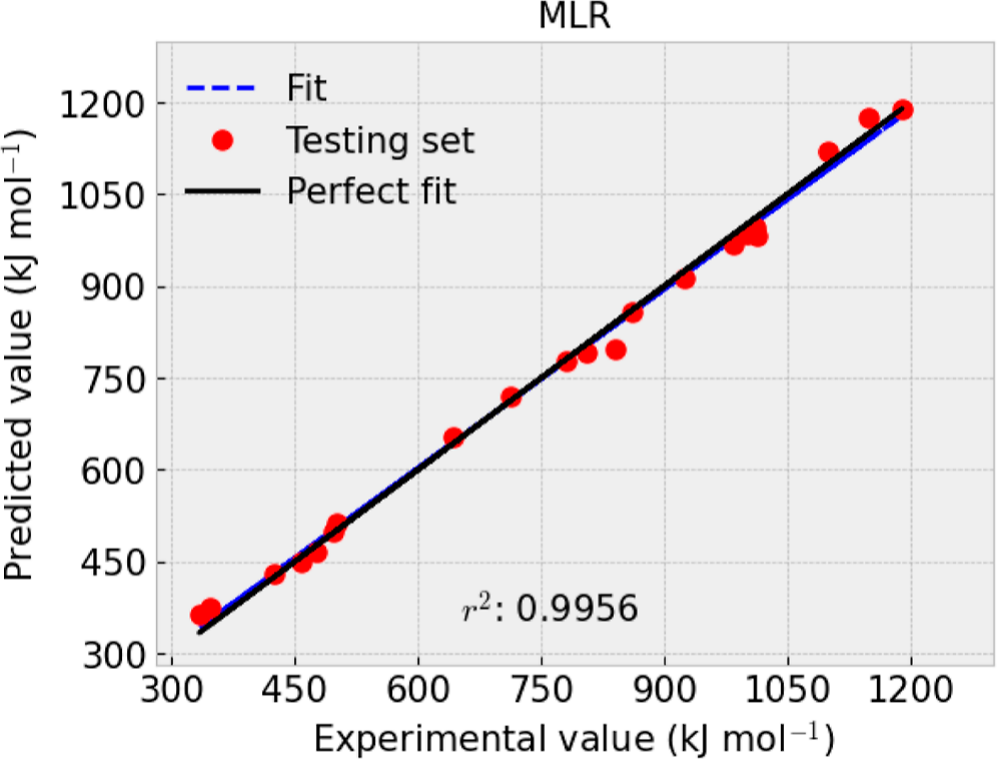
–Δ_f_*H*_*m*_° (cr, 298.15 K) experimental and predicted value fitting of using MLR.

The subroutine “train_test_split” from Python Scikit-learn package was used for the data set random division, the random_state used for the training division and test set, were seeds 98 and 832 for the gas phase and crystalline phase, respectively, and in order to fix the randomness on the data division, some considerations (evaluation metrics) were taken, such as the coefficient of determination (*R*^2^), the mean absolute error (MAE), and the root-mean-square error (RMSE). The results obtained for the MLR algorithm used are presented in Figures 1–4 and Table 9.

**Table 9:** Evaluation Metrics for the MLR Algorithm

gas phase								
	train	test		train	test		train	test
*R*^2^	0.9994	0.9986	MAE	3.0576	6.88	RMSE	4.5482	8.05

crystalline phase								
	train	test		train	test		train	test
*R*^2^	0.9994	0.9952	MAE	3.7417	14.9606	RMSE	5.9598	18.5711

Partitioning the data into training and test sets can be detrimental because not all regressor variables can be found in both sets, but when the evaluation metrics obtained for this model are revised, it has been noticed that the training set has correct learning from the data and makes good predictions to the test set.

In Table 10, it can be observed that the MLR algorithm predicts a close value to the experimental one reported in this work, obtaining only a −0.1 difference on the gas phase; on the other hand, its structural isomer prediction in this phase seems to be coherent since the steric hindrance generated by the each molecule acid groups makes the trimesic acid most stable, followed by the TMAc and at last the hemimellitic acid; this affirmation can be verified from obtained enthalpies of formation. Regarding the crystalline phase, the TMAc value reparametrization has been proved to be advantageous, since a smaller error is obtained respect to those reported in the literature. Additionally, using MLR, it is possible to obtain the coefficients value of each parameter within the regression; in other words, the functional groups values proposed by Benson^76^ were reparameterized following the next values. Those were previously reported by Benson and the remeasured values are shown in Table 11. These new values are specific to the carboxylic acid properties prediction, Table S10.

**Table 10:** Predicted Values of Formation Enthalpy in kJ mol^–1^

MLR						
acid	Δ_f_*H*_*m*_°(g)^a^	Δ_f_*H*_*m*_°(g)^b^	Δ	Δ_f_*H*_*m*_°(cr)^a^	Δ_f_*H*_*m*_°(cr)^b^	Δ
TMAc	–1032.6^c^	–1034.8	2.2	–1177.4^c^	–1177.5	0.1
	–1034.9^c^	–1034.8	–0.1	–1179.2^d^	–1177.5	–1.7
Hemimellitic		–1031.6		–1160.4^d^	–1166.3	5.9
Trimesic		–1045.0		–1190.1^d^	–1189.9	–0.2

a Experimental value.

b Predicted value.

c Experimental value of this work.

d Taken on ref (36).

**Table 11:** Update of Benson Functional Groups for Carboxylic Acids in kJ mol^–1^

group	Benson (g)	new (g)	Benson (cr)	new (cr)
CO-(O)(CO)	–123.75	–82.19	–120.81	–108.4
CO-(C_*D*_)(O)	–136.73	18.98	–134.1	
CO-(C)(O)	–137.24	–79.59	–153.6	–101.62
CO-(H)(O)	–124.39	0		
CO-(O)(C_*B*_)	–125.0	–44.64	–145.0	–37.98
O-(H)(CO)	–254.3	–187.44	–282.15	–248.0
O-(C)(C_*B*_)	–92.55	–18.21	–122.87	–6.83
C_*D*_-(H)(CO)	32.3	18.98	7.82	
C_*B*_-(CO)(C_*B*_)_2_	15.5	–44.64	8.15	–37.98
C_*B*_-(O)(C_*B*_)_2_	–4.75	–18.21	1.0	–71.31
C-(CO)(C)_3_	23.93	–24.93	24.02	–75.39
C-(H)_2_(CO)(C)	–21.84	–38.03	–27.9	–55.35
C-(H)_3_(O)	–42.26	–18.21	–46.74	–6.83
C-(H)_3_(C)	–42.26	49.39	–46.74	–4.37
C-(H)_3_(C_*B*_)		45.32	–18.17	
C-(H)_2_(C)_2_	–20.63	–21.15	–29.41	–27.6
C_*D*_-(H)_2_	26.32	18.98	22.43	
C_*B*_-(H)(C_*B*_)_2_	13.81	38.41	6.53	11.13
C_*B*_-(C)(C_*B*_)_2_	23.64	18.91	13.9	18.79
C-(H)_2_(C)(C_*B*_)	–21.34	–26.41	–22.1	0.61
CH_3_(qua)	–4.56	–49.86	–4.35	0.53
O-(H)(C_*B*_)	–160.3		–199.25	–64.48
C-(H)(CO)(C)_2_	–0.25		–9.83	–82.31
C-(H)(C)_3_	–1.17		–5.98	16.73
CH_3_(tert)	–2.26		–2.34	–0.03
O-(H)(C)	–159.33		–199.66	–105.79
C-(H)_2_(CO)_2_	–30.74		–19.1	–74.85
C-(H)(O)(CO)(C)	126.63		–14.39	–105.79
radical 1		–21.59		–5.75
radical 2		–2.19		–5.2
radical 3		–8.74		–9.74
radical 4		–7.27		–8.11
correction 1		0.92		39.51
correction 1 meta		–2.28		–14.2
correction 1 ortho		–14.84		–6.77
correction 1 para		16.19		–18.54
correction 2		34.31		24.82
correction 2 meta		–16.64		–12.74
correction 2 ortho		0		6.62
correction 2 para		–17.67		–18.7
correction 3		6.58		10.03
correction 3 meta		–5.75		–10.55
correction 3 ortho		11.66		20.47
correction 3 para		–12.49		–19.94
correction 4		1.21		2.92
correction 4 meta		0		–9.4
correction 4 ortho		–1.21		6.48

As can be seen in Table 11, the difference between the past and the reparameterized values is notorious in certain functional groups; since it is considered new values and including new ones, it is possible to have a better prediction scenario for future molecules that are still being synthesized today; on the other hand, the additional parameters proposed for this model (corrections for aromaticity) have a weight within the prediction and thus improve it. The presence of all predictor variables (groups or corrections) and their assigned numerical values for each molecule can be clearly observed in Table S11.

## Conclusions

The enthalpies of fusion and vaporization were determined by DSC and thermogravimetric analysis, respectively. The standard enthalpy of formation in the crystalline phase was obtained by combustion calorimetry getting a 1.8 kJ mol^–1^ difference from that obtained by Yukhno et al. The TMAc experimental standard molar enthalpy of formation in gas and crystalline phase showed an excellent agreement with respect to the predicted value from supervised learning algorithms, obtaining 2.2 and 5.9 kJ mol^–1^ maximum difference. Finally, by using the functional groups proposed by Benson, it was possible to create an algorithm capable of enthalpies of formation in both gas and crystalline phases predictions. After the reparameterization, it is evident that some coefficients obtained from the MLR do not have a significant change when compared to those presented by authors such as Benson or Gani. However, for the carboxylic acids specific case, the aromaticity corrections contribution implemented to are for a better prediction on the parameters. The use of supervised learning in enthalpy prediction sets the tone for the exploration of new techniques in computational chemistry fitted to the experimental data.

## Supporting Information Available

In addition to MLR, other supervised learning methods were tested, these methods as well as the data sets to verify the results obtained can be found at the GitHub repository: https://github.com/FDS116/TMAc.git. The data set consists of values obtained experimentally, subsequently taken to the standard state, and 298.15 K, taken from different references. In the case of MLR, the “random_state” of 98 was used for the gas phase and 832 for the crystalline phase. Google Colab is a freely available software at the following link: https://colab.research.google.com. All images mentioned in the paper (ZIP)
TGA thermogram, diffractogram of TMAc, thermogram of indium obtained in DSC, thermogravimetric data, comparison of vaporization and sublimation enthalpies, comparison of sublimation enthalpies of pyrene, experimental values of C_p,m_(cr) of TMAc, linear regression of TMAc data, calculation of formation enthalpy, and group reparametrization (PDF)
