# Supplementary figures and images for: Experimental Determination of the Standard Enthalpy of Formation of Trimellitic Acid and Its Prediction by Supervised Learning
Source: J Phys Chem A. 2024 Mar 6;128(11):2200–9. doi: 10.1021/acs.jpca.3c05235 (PMC10961834; doi:10.1021/acs.jpca.3c05235)

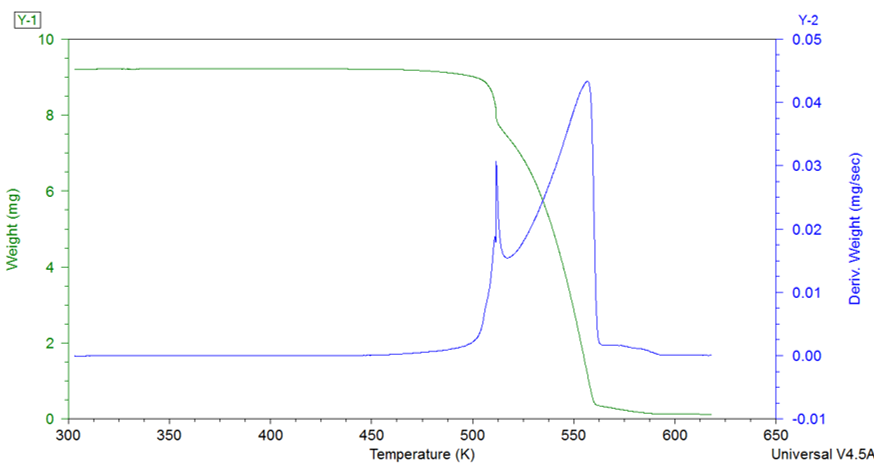

Supplement: Supplementary file 1 — jp3c05235_si_001.zip [file jp3c05235_si_001.zip › Fig S-1.png]

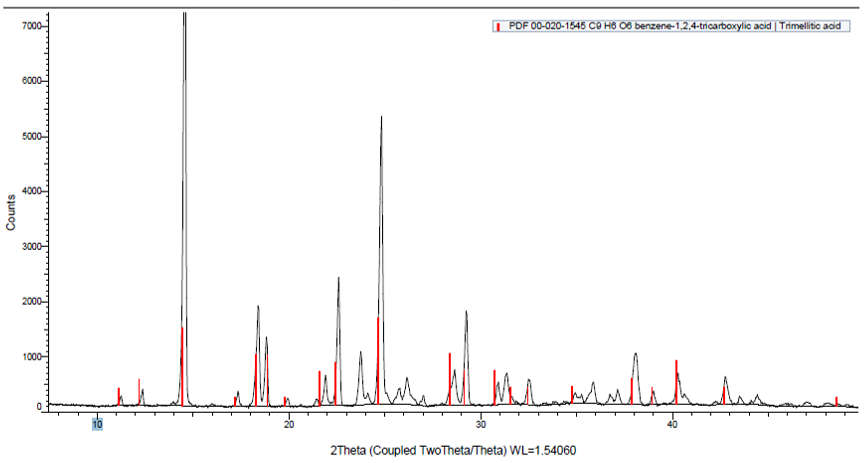

Supplement: Supplementary file 1 — jp3c05235_si_001.zip [file jp3c05235_si_001.zip › Fig S-2.png]

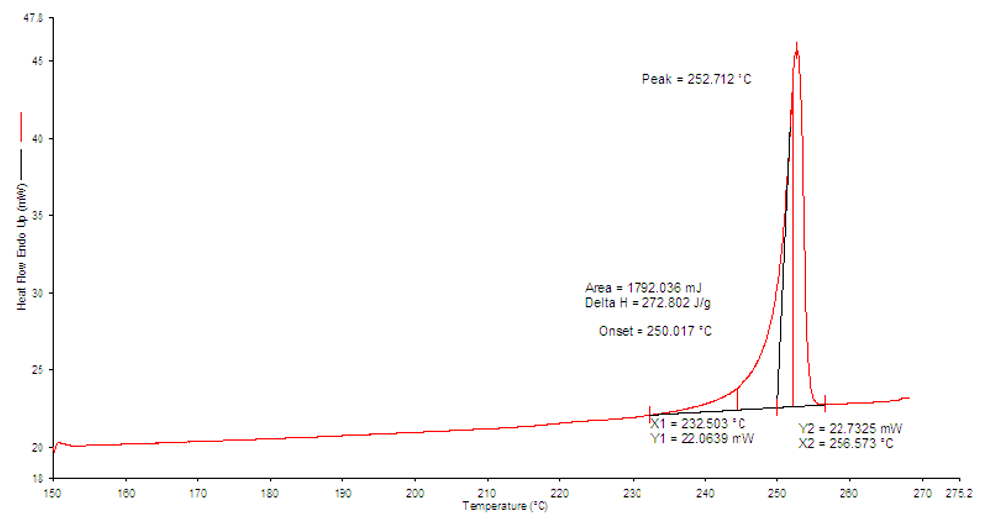

Supplement: Supplementary file 1 — jp3c05235_si_001.zip [file jp3c05235_si_001.zip › Fig S-3.png]

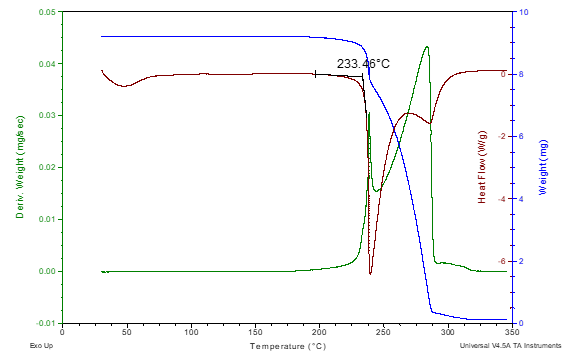

Supplement: Supplementary file 1 — jp3c05235_si_001.zip [file jp3c05235_si_001.zip › Fig S-4.png]

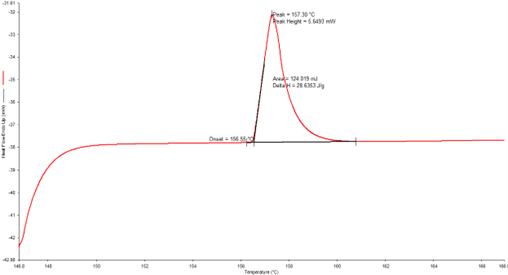

Supplement: Supplementary file 1 — jp3c05235_si_001.zip [file jp3c05235_si_001.zip › Fig S-5.png]

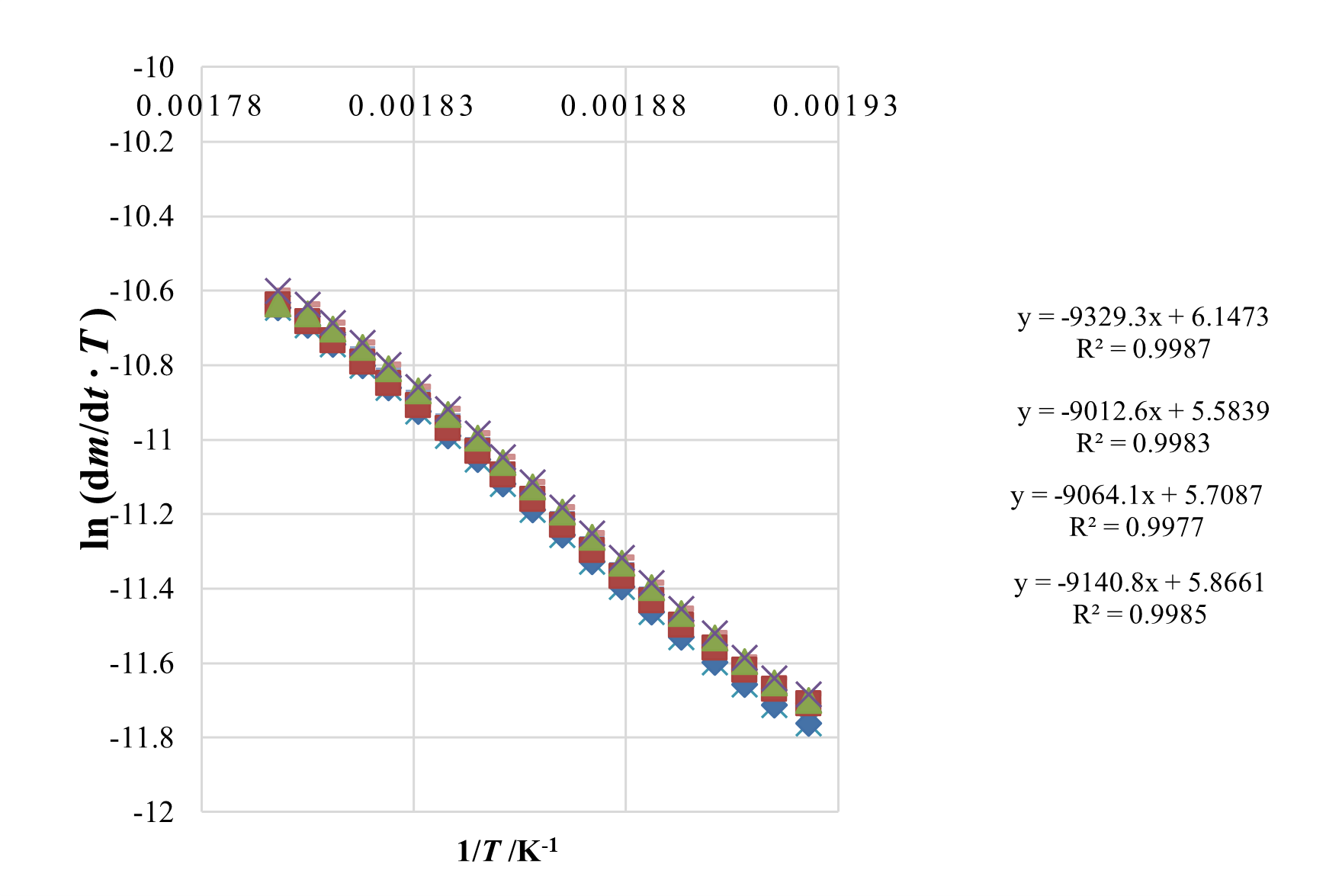

Supplement: Supplementary file 1 — jp3c05235_si_001.zip [file jp3c05235_si_001.zip › Fig S-6.png]

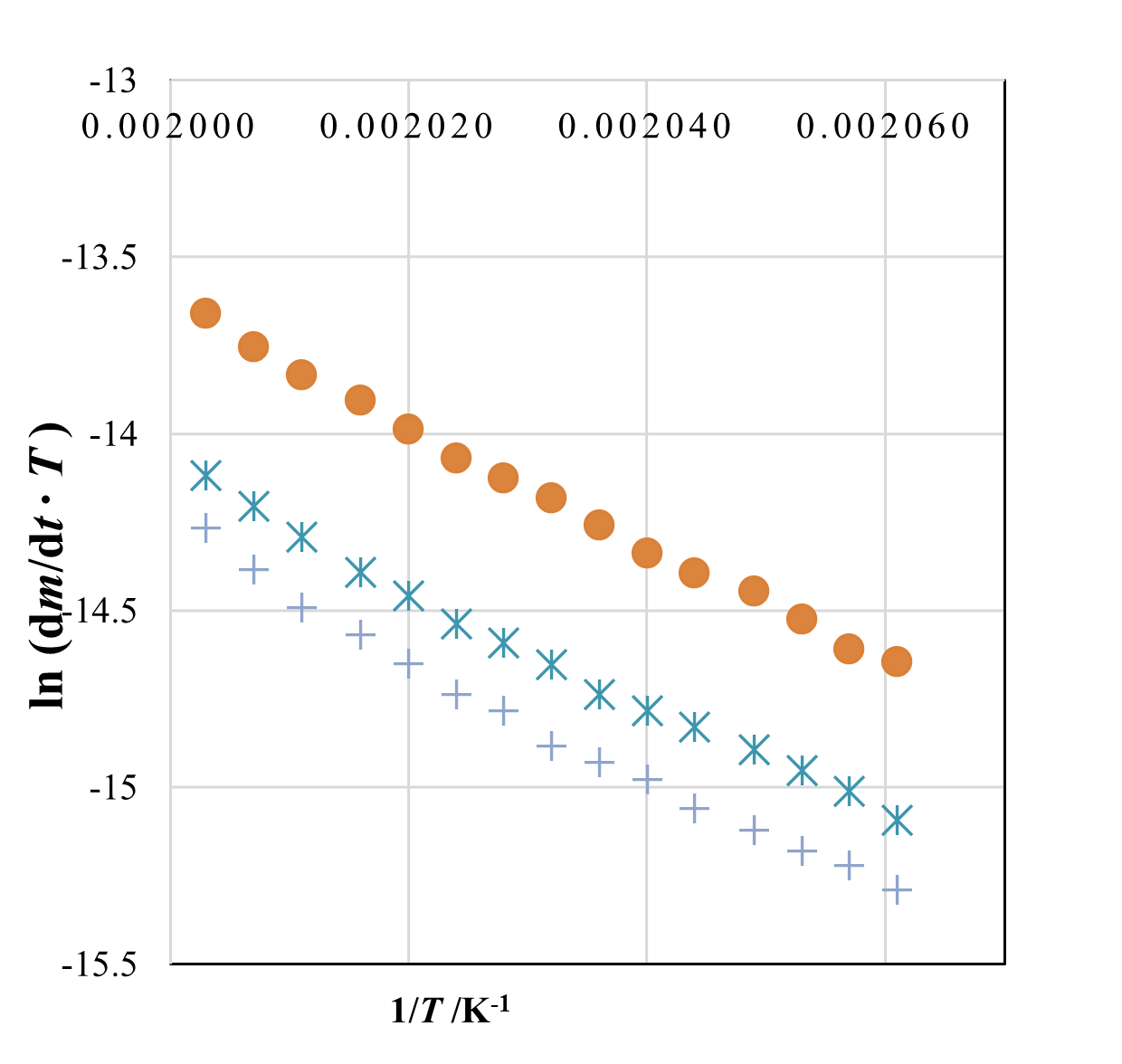

Supplement: Supplementary file 1 — jp3c05235_si_001.zip [file jp3c05235_si_001.zip › Fig S-7.png]

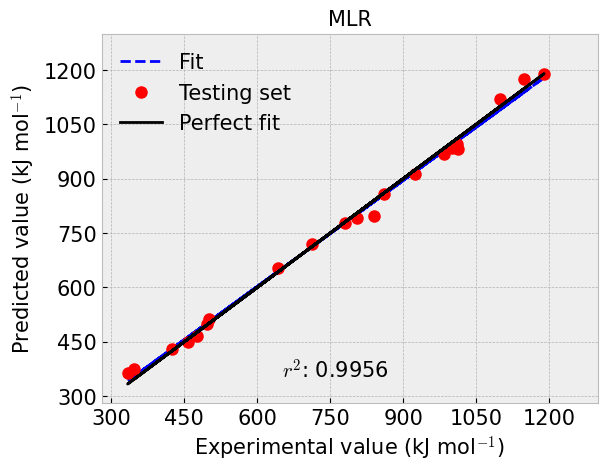

Supplement: Supplementary file 1 — jp3c05235_si_001.zip [file jp3c05235_si_001.zip › MLR_cr_test.png]

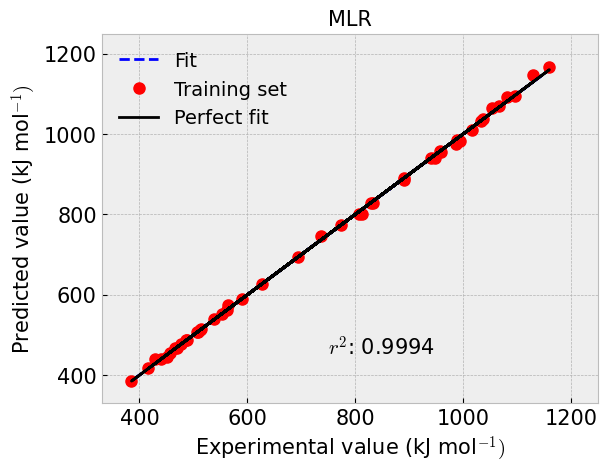

Supplement: Supplementary file 1 — jp3c05235_si_001.zip [file jp3c05235_si_001.zip › MLR_cr_train.png]

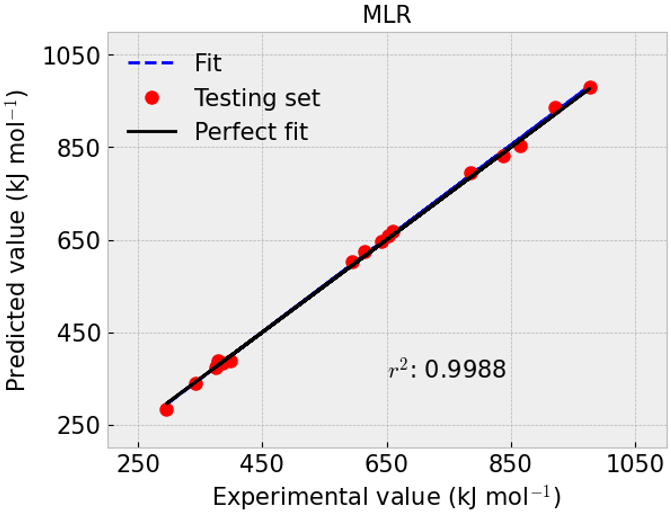

Supplement: Supplementary file 1 — jp3c05235_si_001.zip [file jp3c05235_si_001.zip › MLR_gas_test.png]

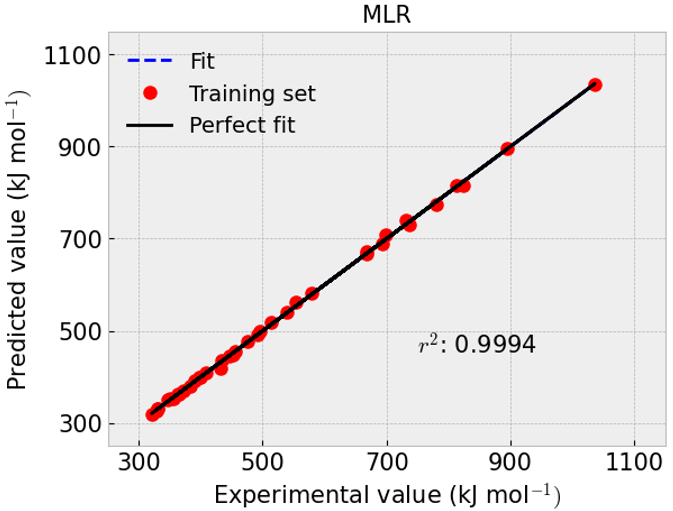

Supplement: Supplementary file 1 — jp3c05235_si_001.zip [file jp3c05235_si_001.zip › MLR_gas_train.png]

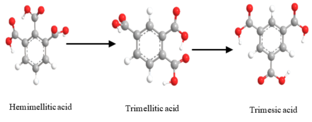

Supplement: Supplementary file 1 — jp3c05235_si_001.zip [file jp3c05235_si_001.zip › TOC graphic.png]
