# Supplementary material for: Experimental Determination of the Standard Enthalpy of Formation of Trimellitic Acid and Its Prediction by Supervised Learning
Source: J Phys Chem A. 2024 Mar 6;128(11):2200–9. doi: 10.1021/acs.jpca.3c05235 (PMC10961834; doi:10.1021/acs.jpca.3c05235)
Supplement: Supplementary file 2 — jp3c05235_si_002.pdf [file jp3c05235_si_002.pdf]

Experimental Determination of the Standard Enthalpy of Formation of Trimellitic Acid and its Prediction by Supervised Learning

Fausto Díaz-Sánchez,† Miguel Angel García-Castro,*,† María Patricia Amador-Ramírez,‡ Jesús Andrés Arzola-Flores,† and Ximena Limón Aguilar†

†*Facultad de Ingeniería Química de la Benemérita Universidad Autónoma de Puebla, 18 Sur y Av. San Claudio, C.P. 72570, Puebla Pue, Mexico*

‡*Facultad de Ciencias Químicas de la Benemérita Universidad Autónoma de Puebla, 14 Sur y Av. San Claudio, C.P. 72570, Puebla Pue, Mexico*

E-mail: miguel.garciacastro@correo.buap.mx

Supporting Information Available

LIST OF CONTENTS

Figure S1: TGA thermogram of TMAc

Figure S2: Diffractogram of TMAc

Figure S3: Example of Thermogram of TMAc, obtained in DSC using gold crucibles and mass around 6 mg

Figure S4: Example of thermogram of TMAc, obtained by SDT. In these experiments was used an alumina crucible and the values of mass were around 9.0 mg. The red line corresponds to DSC signal. The little peak observed near of 50 °C corresponds to a stabilization period of the DSC signal (these peak is observed in all thermograms, included to the reference

compounds)

Figure S5: Thermogram of Indium obtained in DSC using gold capsules

Table S1: Thermogravimetric data of Pyrene to determine vaporization enthalpy

Table S2: Thermogravimetric data of Phenanthrene to determine vaporization enthalpy

Table S3: Thermogravimetric data of Pyrene to determine sublimation enthalpy

Table S4: Thermogravimetric data of Anthracene to determine sublimation enthalpy

Table S5: Comparison of vaporization and sublimation enthalpies for standard compounds

Table S6: Comparison of sublimation enthalpies of pyrene at T=298.15 K calculated by two routes

Table S7: Experimental values of $C_{p,m}$(cr) of TMAc at different temperatures

Table S8: Thermogravimetric data of TMAc to determine vaporization enthalpy

Figure S6: Linear regression of TMAc data as function of $1/T$ to determinate the enthalpy of vaporization, $\Delta_l^g H_m(T_m)$

Table S9: Thermogravimetric data of TMAc to determine sublimation enthalpy

Figure S7: Linear regression of TMAc data as function of $1/T$ to determinate the enthalpy of sublimation, $\Delta_{cr}^g H_m(T_m)$

Table S10: Calculation of formation enthalpy in kJ mol^{-1}.

Table S11: Group reparametrization

In Figure S1, obtained by thermogravimetric analysis, the thermogram of TMAc purified is disclosed. As shown in figure, at 373 K there is no mass loss due to the vaporization of water.

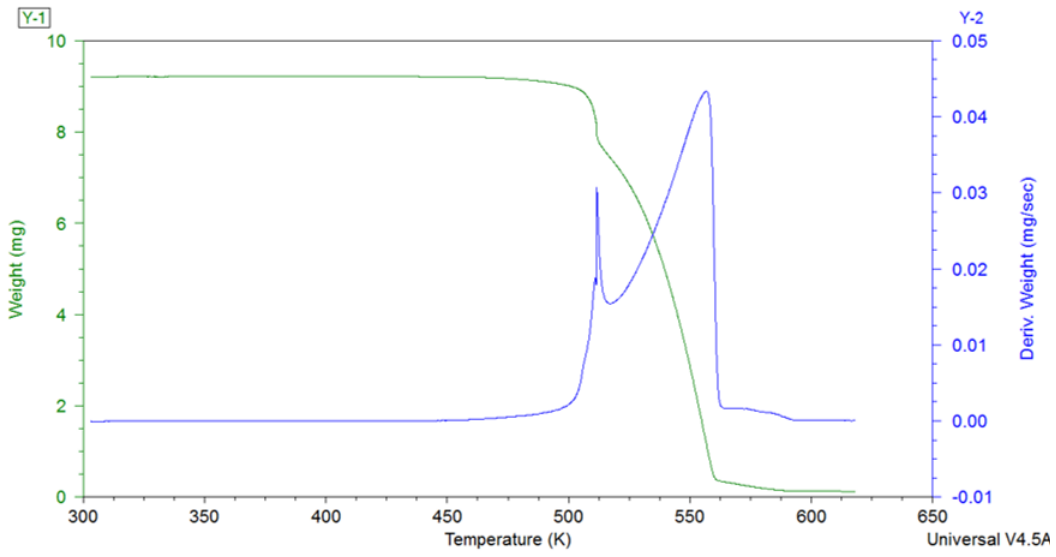

Figure S1: TGA thermogram of TMAc.

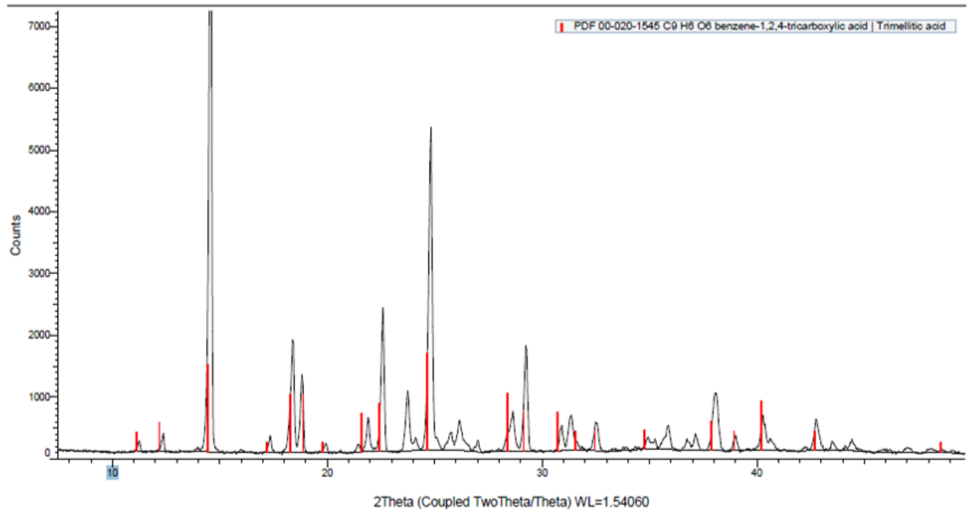

Figure S2: Diffractogram of TMAc.

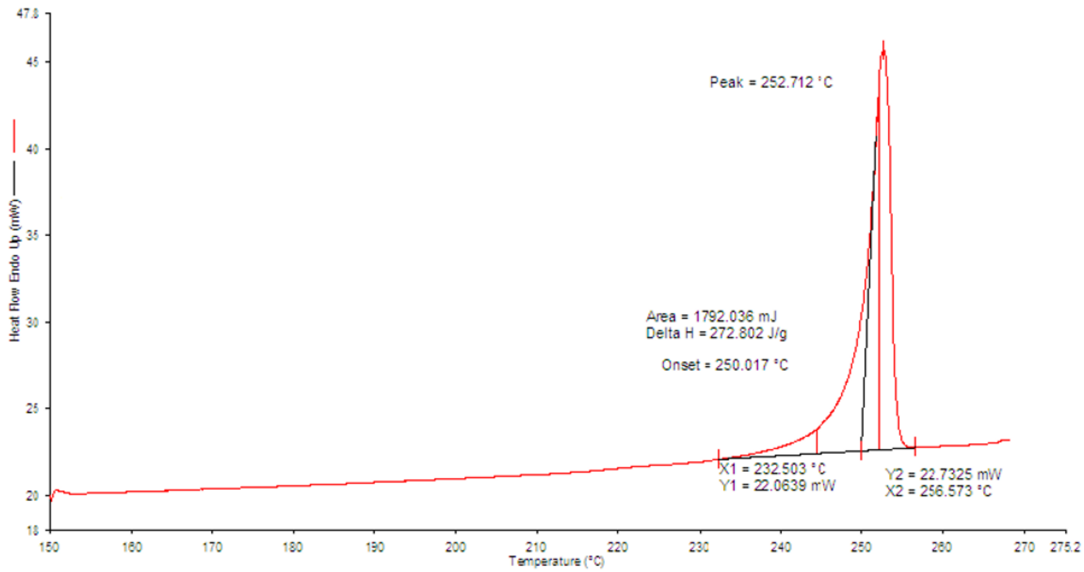

Figure S3: Example of Thermogram of TMAc, obtained in DSC using gold crucibles and mass around 6 mg.

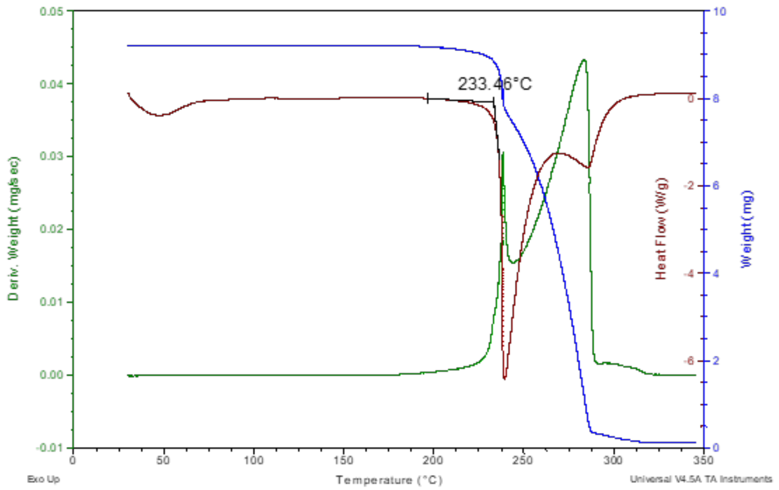

Figure S4: Example of thermogram of TMAc, obtained by SDT. In these experiments was used an alumina crucible and the values of mass were around 9.0 mg. The red line corresponds to DSC signal. The little peak observed near of 50 °C corresponds to a stabilization period of the DSC signal (this peak is observed in all thermograms, included to the reference compounds).

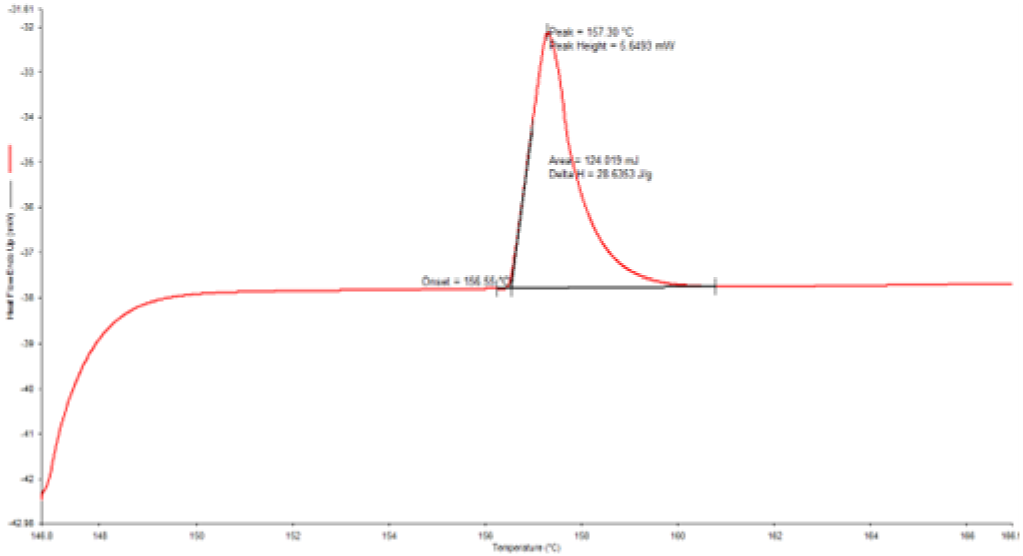

Figure S5: Thermogram of Indium obtained in DSC using gold capsules.

Table S1: Thermogravimetric data of Pyrene to determine vaporization enthalpy

T	m	$dm/dt\cdot 10^9$	$1/T\cdot 10^3$	ln $(dm/dt)\cdot$T
K	mg	kg$\cdot$ s^{-1}	K^{-1}	
Series 1				
430.15	10.0522	0.522	2.325	-15.3094
435.15	10.0360	0.659	2.298	-15.0640
440.15	10.0134	0.827	2.272	-14.8256
445.15	9.9871	1.056	2.246	-14.5704
450.15	9.9502	1.331	2.221	-14.3278
ln $(dm/dt\cdot T) = 6.65 - 9447.3/T$, r$^2 = 0.9997$, $\sigma_m = 97.14$, $\sigma_y = 0.22$				
$\Delta_l^g H_m^o(440.15\text{K}) = (78.5 \pm 0.8)^{\text{a}}$ kJ $\cdot$ mol^{-1}				
Series 2				
430.15	8.9326	0.544	2.325	-15.2679
435.15	8.9161	0.711	2.298	-14.9887

Continued on next page

Table S1 – continued from previous page

T	m	$dm/dt\cdot 10^9$	$1/T\cdot 10^3$	ln $(dm/dt\cdot$T)
K	mg	kg$\cdot$ s^{-1}	K^{-1}	
440.15	8.8925	0.888	2.272	-14.7549
445.15	8.8626	1.124	2.246	-14.5080
450.15	8.8259	1.416	2.221	-14.2658
ln$(dm/dt \cdot T)$ = 6.96 - 9558.51/T, r^2 = 0.9996, σ_m = 110.81, σ_y = 0.25				
$\Delta_l^g H_m^o$(440.15K) = (79.5 ± 0.9)[a] kJ$\cdot$ mol^{-1}				
Series 3				
430.15	9.4629	0.556	2.325	-15.2679
435.15	9.4433	0.556	2.325	-14.9887
440.15	9.4213	0.908	2.272	-14.7549
445.15	9.3904	1.147	2.246	-14.5080
450.15	9.3531	1.439	2.221	-14.2658
ln$(dm/dt \cdot T)$ = 6.96 - 9549.48/T, r^2 = 0.9999, σ_m = 57.67, σ_y = 0.13				
$\Delta_l^g H_m^o$(440.15K) = (79.4 ± 0.5)[a] kJ$\cdot$ mol^{-1}				
Weighted average value <$\Delta_l^g H_m^o$(440.15K) = (79.2 ± 0.7)>[b] kJ$\cdot$ mol^{-1}				
<$\Delta_l^g H_m^o$(298.15K) = (91.4 ± 1.4)>[c] kJ$\cdot$ mol^{-1}				

[a] The uncertainty for each enthalpy of vaporization value was calculated with standard deviation of the slope, which is a standard uncertainty. [b] The weighted average value μ and its standard deviation σ, were calculated as $\mu = \sum(x_i/\sigma_i^2)/\sum(1/\sigma_i^2)$ and σ^2= N/$\sum(1/\sigma_i^2)$, where x$_i$ is each of the N vaporization enthalpy data and its respective standard deviation σ_i. The experiments of vaporization were realized under average atmospheric pressure (78.8 kPa), u(P)= 1 kPa. The uncertainty corresponds to combined uncertainty. [c] The standard molar enthalpy of vaporization was obtained by equations 11, 12 and 13 where $C_{p,m}$(g) = 204.2 J$\cdot$ mol$^{-1}\cdot$ K^{-1} according to Rojas et al.[i] [i] Rojas, A.; Orozco, E. Measurement of the enthalpies of vaporization and sublimation of solids aromatic hydrocarbons by differential scanning

calorimetry. *Thermochim.Acta*, 2003, 405, 93-107. doi:10.1016/S0040-6031(03)00139-4

Table S2: Thermogravimetric data of Phenanthrene to determine vaporization enthalpy

T	m	$dm/dt \cdot 10^9$	$1/T \cdot 10^3$	ln $(dm/dt \cdot \mathrm{T})$
K	mg	kg$\cdot$ s^{-1}	K^{-1}	
		Series 1		
378.15	11.0130	0.252	2.644	-16.1663
383.15	11.0052	0.271	2.610	-16.0801
388.15	10.9959	0.404	2.576	-15.6677
393.15	10.9836	0.546	2.544	-15.3551
398.15	10.9642	0.699	2.512	-15.0941
403.15	10.9414	0.896	2.480	-14.8340
408.15	10.9099	1.169	2.450	-14.5555
413.15	10.8697	1.506	2.420	-14.2900
418.15	10.8188	1.886	2.391	-14.0530
423.15	10.7531	2.396	2.363	-13.8017
		$\ln(dm/dt \cdot T)$ = 6.83 - 8729.66/T, r^2 = 0.9961, σ_m = 192.28, σ_y = 0.48		
		$\Delta_l^g H_m^o$(400.65K) = (72.6 ± 1.6)[a] kJ$\cdot$ mol^{-1}		
		Series 2		
378.15	11.2410	0.212	2.644	-16.3391
383.15	11.2334	0.307	2.610	-15.9557
388.15	11.2257	0.370	2.576	-15.7561
393.15	11.2124	0.476	2.544	-15.4914
398.15	11.1977	0.637	2.512	-15.1874
403.15	11.1745	0.832	2.480	-14.9079
408.15	11.1455	1.073	2.450	-14.6412

Continued on next page

Table S2 – continued from previous page

T	m	$dm/dt \cdot 10^9$	$1/T \cdot 10^3$	ln $(dm/dt \cdot \mathrm{T})$
K	mg	kg· s^{-1}	K^{-1}	
413.15	11.1086	1.381	2.420	-14.3766
418.15	11.0645	1.753	2.391	-14.1261
423.15	11.0050	2.221	2.363	-13.8776
$\ln(dm/dt \cdot T)$ = 6.59 - 8664.41/T, $r^2 = 0.9988$, $\sigma_m = 107.97$, $\sigma_y = 0.27$				
$\Delta_l^g H_m^o(400.65\mathrm{K}) = (72.0 \pm 0.9)^{a}$ kJ· mol^{-1}				
Series 3				
378.15	11.2512	0.230	2.644	-16.2577
383.15	11.2438	0.286	2.610	-16.0266
388.15	11.2322	0.390	2.576	-15.7035
393.15	11.2205	0.519	2.544	-15.4049
398.15	11.2022	0.669	2.512	-15.1384
403.15	11.1802	0.881	2.480	-14.8507
408.15	11.1496	1.147	2.450	-14.5745
413.15	11.1103	1.460	2.420	-14.3210
418.15	11.0600	1.874	2.391	-14.0594
423.15	10.9963	2.363	2.363	-13.8156
$\ln(dm/dt \cdot T)$ = 7.00 - 8811.62/T, $r^2 = 0.9996$, $\sigma_m = 64.40$, $\sigma_y = 0.16$				
$\Delta_l^g H_m^o(400.65\mathrm{K}) = (73.3 \pm 0.5)^{a}$ kJ· mol^{-1}				
Weighted average value $<\Delta_l^g H_m^o(400.65\mathrm{K}) = (73.0 \pm 0.7)>^{b}$ kJ· mol^{-1}				
$<\Delta_l^g H_m^o(298.15\mathrm{K}) = (81.2 \pm 1.4)>^{c}$ kJ· mol^{-1}				

[a] The uncertainty for each enthalpy of vaporization value was calculated with standard deviation of the slope, which is a standard uncertainty. [b] The weighted average value μ and its standard deviation σ, were calculated as $\mu = \sum(x_i/\sigma_i^2)/\sum(1/\sigma_i^2)$ and σ^2 =N/$\sum(1/\sigma_i^2)$], where x_i is each of the N vaporization

enthalpy data and its respective standard deviation σ_i. The experiments of vaporization were realized under average atmospheric pressure (78.8 kPa), u(P)= 1 kPa. The uncertainty corresponds to combined uncertainty. [c] The standard molar enthalpy of vaporization was obtained by equations 11, 12 and 13 where $C_{p,m}$(g) = 185.7 J· mol^{-1}· K^{-1} according to Rojas et al.[i]

[i] Rojas, A.; Orozco, E. Measurement of the enthalpies of vaporization and sublimation of solids aromatic hydrocarbons by differential scanning calorimetry. *Thermochim.Acta*, 2003, 405, 93-107. doi:10.1016/S0040-6031(03)00139-4

Table S3: Thermogravimetric data of Pyrene to determine sublimation enthalpy

T	m	$dm/dt\cdot 10^9$	$1/T\cdot 10^3$	ln ($dm/dt\cdot$T)
K	mg	kg· s^{-1}	K^{-1}	
Series 1				
370.15	12.8614	0.051	2.702	-17.7853
375.15	12.8598	0.089	2.666	-17.2151
380.15	12.8568	0.114	2.631	-16.9543
385.15	12.8522	0.183	2.596	-16.4679
390.15	12.8455	0.270	2.563	-16.0661
395.15	12.8358	0.369	2.531	-15.7410
ln($dm/dt\cdot T$) = 14.18 - 11811.73T, r^2 = 0.9943, σ_m = 447.71, σ_y = 1.17				
$\Delta_l^g H_m^o$(382.65K) = (98.2 ± 3.7)^a kJ· mol^{-1}				
Series 2				
370.15	12.4414	0.052	2.702	-17.7659
375.15	12.4390	0.077	2.666	-17.3599
380.15	12.4364	0.109	2.631	-16.9991
385.15	12.4318	0.194	2.596	-16.4095
390.15	12.4250	0.252	2.563	-16.1351

Continued on next page

Table S3 – continued from previous page

T	m	$dm/dt\cdot 10^9$	$1/T\cdot 10^3$	ln $(dm/dt\cdot \mathrm{T})$
K	mg	kg$\cdot$ s^{-1}	K^{-1}	
395.15	12.4157	0.356	2.531	-15.7768
$\ln(dm/dt \cdot T)$ = 14.27 - 11858.66/T, r^2 = 0.9940, σ_m = 458.85, σ_y = 1.20				
$\Delta_l^g H_m^o$(382.65K) = (98.6 ± 1.9)[a] kJ$\cdot$ mol^{-1}				
Series 3				
370.15	9.0839	0.050	2.702	-17.8051
375.15	9.0798	0.084	2.666	-17.2729
380.15	9.0735	0.127	2.631	-16.8463
385.15	9.0645	0.180	2.596	-16.4844
390.15	9.0518	0.260	2.563	-16.1038
395.15	9.0335	0.364	2.531	-15.7546
$\ln(dm/dt \cdot T)$ = 14.11 - 11788.97/T, r^2 = 0.9964, σ_m = 352.94, σ_y = 0.92				
$\Delta_l^g H_m^o$(382.65K) = (98.0 ± 3.0)[a] kJ$\cdot$ mol^{-1}				
Weighted average value <$\Delta_l^g H_m^o$(382.65K) = (98.4 ± 2.6)>[b] kJ$\cdot$ mol^{-1}				
<$\Delta_l^g H_m^o$(298.15K) = (101.1 ± 5.2)>[c] kJ$\cdot$ mol^{-1}				

[a] The uncertainty for each enthalpy of vaporization value was calculated with standard deviation of the slope, which is a standard uncertainty. [b] The weighted average value μ and its standard deviation σ, were calculated as $\mu = \sum(x_i/\sigma_i^2)/\sum(1/\sigma_i^2)$ and σ^2 =N/$\sum(1/\sigma_i^2)$], where x_i is each of the N vaporization enthalpy data and its respective standard deviation σ_i. The experiments of vaporization were realized under average atmospheric pressure (78.8 kPa), u(P)= 1 kPa. The uncertainty corresponds to combined uncertainty. [c] The standard molar enthalpy of vaporization was obtained by equations 11, 12 and 13 where $C_{p,m}$(g) = 204.2 J$\cdot$ mol$^{-1}\cdot$ K^{-1} according to Rojas et al.[i] [i] Rojas, A.; Orozco, E. Measurement of the enthalpies of vaporization and sublimation of solids aromatic hydrocarbons by differential scanning calorimetry. *Thermochim.Acta*, 2003, 405, 93-107. doi:10.1016/S0040-6031(03)00139-4

Table S4: Thermogravimetric data of Anthracene to determine sublimation enthalpy

T	m	$dm/dt \cdot 10^9$	$1/T \cdot 10^3$	ln $(dm/dt \cdot \mathrm{T})$
K	mg	kg$\cdot$ s^{-1}	K^{-1}	
		Series 1		
380.15	15.9043	0.169	2.631	-16.5606
385.15	15.8979	0.270	2.596	-16.0790
390.15	15.8883	0.395	2.563	-15.6856
395.15	15.8748	0.568	2.531	-15.3096
400.15	15.8545	0.773	2.499	-14.9889
405.15	15.8240	1.071	2.468	-14.6504
$\ln(dm/dt \cdot T)$ = 14.03 - 11608.74T, r^2 = 0.9971, σ_m = 310.67, σ_y = 0.79				
$\Delta_l^g H_m^o$(392.65K) = (96.5 ± 2.6)^a kJ$\cdot$ mol^{-1}				
		Series 2		
380.15	17.9838	0.176	2.631	-16.5200
385.15	17.9770	0.283	2.596	-16.0319
390.15	17.9673	0.406	2.563	-15.6581
395.15	17.9529	0.563	2.531	-15.3185
400.15	17.9325	0.812	2.499	-14.9397
405.15	17.9036	1.146	2.468	-14.5827
$\ln(dm/dt \cdot T)$ = 14.30 - 11696.94/T, r^2 = 0.9984, σ_m = 231.44, σ_y = 0.59				
$\Delta_l^g H_m^o$(392.65K) = (97.2 ± 1.9)^a kJ$\cdot$ mol^{-1}				
		Series 3		
380.15	7.7741	0.200	2.631	-16.3921
385.15	7.7661	0.320	2.596	-15.9091
390.15	7.7548	0.471	2.563	-15.5096

Continued on next page

Table S4 – continued from previous page

T	m	$dm/dt \cdot 10^9$	$1/T \cdot 10^3$	ln $(dm/dt \cdot \mathrm{T})$
K	mg	kg· s^{-1}	K^{-1}	
395.15	7.7384	0.660	2.531	-15.1595
400.15	7.7148	0.939	2.499	-14.7944
405.15	7.6821	1.302	2.468	-14.4551

$\ln(dm/dt \cdot T)$ = 14.61 - 11766.80/T, r^2 = 0.9981, σ_m = 257.23, σ_y = 0.66

$\Delta_l^g H_m^o$(392.65K) = (97.8 ± 2.1)[a] kJ· mol^{-1}

Weighted average value <$\Delta_l^g H_m^o$(392.65K) = (97.2 ± 2.1)>[b] kJ· mol^{-1}

<$\Delta_l^g H_m^o$(298.15K) = (100.2 ± 4.2)>[c] kJ· mol^{-1}

[a] The uncertainty for each enthalpy of vaporization value was calculated with standard deviation of the slope, which is a standard uncertainty. [b] The weighted average value μ and its standard deviation σ, were calculated as $\mu = \sum(x_i/\sigma_i^2)/\sum(1/\sigma_i^2)$ and σ^2 =N/$\sum(1/\sigma_i^2)$], where x$_i$ is each of the N vaporization enthalpy data and its respective standard deviation σ_i. The experiments of vaporization were realized under average atmospheric pressure (78.8 kPa), u(P)= 1 kPa. The uncertainty corresponds to combined uncertainty. [c] The standard molar enthalpy of sublimation was obtained by equation 15 according to Chickos et al.[i] [i]Chickos, J.S.; Hosseini, S.; Hesse, D.G.; Liebman, J.F. Heat capacity corrections to a standard state: a comparison of new and some literature methods for organic liquids and solids, *Struct.Chem.*, 1993, 4, 271–278. https://doi.org/10.1007/ BF00673701.

Table S5: Comparison of vaporization and sublimation enthalpies for standard compounds

$\Delta_\alpha^\beta H_m(T_m/K)$	*Interval of T*	T_m	$\Delta_\alpha^\beta H_m^o$(298.15K)	Method[b]	Reference[c]
kJ· mol^{-1}	K	K	kJ· mol^{-1}		
		vaporizationenthalpyofpyrene			
92.4 ± 1.0	298	298	92.4 ± 1.0	CGC	1
81.5 ± 0.8	425-445	435	90.3 ± 1.6	TGA	2

NA	NA	NA	89.4 ± 3.1		3
79.2 ± 0.7	430.15-450.15	440.15	91.4 ± 1.4[a]	TGA	This work
		sublimationenthalpyofpyrene			
97.8 ± 3.3	322-381	352	99.7 ± 3.4	ME	4
97.9 ± 0.6	314-454	384	100.7 ± 2.8	T	5
94.0 ± 0.4	398-423	411	98.4 ± 1.9	IPG	6
97.5 ± 0.7	348-419	384	100.3 ± 2.0	ME	7
97.7 ± 0.3	384	384	100.5 ± 1.9	ME	7
98.4 ± 2.6	370.15-395.15	382.65	101.1 ± 5.2[a]	TGA	This work
		sublimationenthalpyofanthracene			
96.3 ± 1.2	380-400	390	99.3 ± 2.4	TGA	2
98.5 ± 3.3	288-323	306	98.8 ± 3.3	ME	4
98.8 ± 0.4	340-360	350	100.2 ± 0.4	ME	8
NA	NA	453	100.8 ± 1.7	DC	9
94.5	423-488	456	99.6	T	10
97.3	329-373	351	99.0	ME	11
97.2 ± 2.1	380.15-405.15	392.65	100.2± 4.2[a]	TGA	This work
		vaporizationenthalpyofPhenanthrene			
NA	NA	NA	79.0 ± 2.4	GC	12
78.7 ± 1.1	298	298	78.7 ± 1.1	CGC	13-16
60.8 ± 0.3	507-545	526	79.8 ± 2.0	I	17
61.8 ± 1.5	505-538	522	80.4 ± 3.9	I	18
73.0 ± 0.7	378.15-423.15	400.65	81.2 ± 1.4[a]	TGA	This work

[a] The uncertainty corresponds to twice the standard combined.

[b] Method: Correlation of Gas Chromatography (CGC); Thermogravimetry (TGA); Gas Chromatography (GC); Inclined pistón gauge (IPG); Method of effusion (ME); Transpiration (T); Static Isoteniscope (I);

Drop Calorimeter (DC).

[c] Data provided in the references:

1. Hanshaw, W.; Nutt, M.; Chickos, J. S. Hypothetical Thermodynamic Properties. Subcooled Vaporization Enthalpies and Vapor Pressures of Polyaromatic Hydrocarbons. *J.Chem.Eng.Data* 2008, 53, 1903. https://doi.org/10.1021/je800300x

2. Ramos, F.; Ledo, J.M.; Flores, H.; Camarillo, E.A.; Carvente, J.; Amador, M.P. Evaluation of sublimation enthalpy by thermogravimetry: Analysis of the diffusion effects in the case of methyl and phenyl substituted hydantoins. *Thermochim.Acta*, 2017, 655, 181-193. https://doi.org/10.1016/j.tca.2017.06.024

3. Roux, M.V.; Temprado, M.; Chickos, J.S.; Nagano, Y. Critically evaluated thermochemical properties of polycyclic aromatic hydrocarbons. *J.Phys.Chem.Ref.Data*, 2008, 37, 1855–1996. https://doi.org/10.1063/1.2955570

4. Goldfarb, J.L.; Suuberg, E.M. Vapor pressures and enthalpies of sublimation of ten polycyclic aromatic hydrocarbons determined via the Knudsen effusion method. *J.Chem.Eng.Data*, 2008, 53, 670-676. https://doi.org/10.1021/je7005133

5. Nass, K.; Lenoir, D.; Kettrup, A. Calculation of the thermodynamic properties of polycyclic aromatic hydrocarbons by an incremental procedure, *Angew.Chem.Int.Ed.Engl.* 1995, 34, 1735–1736. https://doi.org/10.1002/anie.199517351

6. Smith, N.K.; Stewart R.C. Jr.; Osborn, A.G.; Scott, D.W.; Pyrene: Vapor pressure, enthalpy of combustion, and chemical thermodynamic properties. *J.Chem.Thermodyn.* 1980, 12, 919–926. https://doi.org/10.1016/0021-9614(80)90132-9

7. Malaspina, L.; Bardi, G.; Gigli, R. Simultaneous determination by knudsen-effusion microcalorimetric technique of the vapor pressure and enthalpy of vaporization of pyrene and 1, 3, 5-triphenylbenzene. *J.Chem.Thermodyn.* 1974, 6, 1053-1064. https://doi.org/10.1016/0021-9614(74)90067-6

8. Ribeiro da Silva, M.A.V.; Monte, M.J.S.; Santos, L.M.N.B.F. The design, construction, and testing of a new Knudsen effusion apparatus. *J.Chem.Thermodyn.* 2006, 38, 778-787. https://doi.org/10.1016/j.jct.2005.08.013

9. Santos, L.M.N.B.F.; Schroeder, B.; Fernandes, O.O.P.; Ribeiro da Silva, M.A.V. Measurement of enthalpies of sublimation by drop method in a Calvet type calorimeter: design and test of a new system. *Thermochim.Acta* 2004, 415, 15-20. https://doi.org/10.1016/j.tca.2003.07.016

10. Emmenegger, F.; Piccand, M. Vapour pressure measurements with a thermobalance. *J.ThermAnal.Calorim.* 1999, 57, 235-240. https://doi.org/10.1023/a:1010100531350

11. Taylor, J.W.; Crookes, R.J. Vapour pressure and enthalpy of sublimation of 1, 3, 5, 7-tetranitro-1, 3, 5,

7-tetra-azacyclo-octane (HMX). *J.Chem.Soc., FaradayTrans.* 1976, 1, 723-729. https://doi.org/10.1039/F19767200723

12. Haftka, J.J.H.; Parsons, J.R.; Govers, H.A.J. Supercooled liquid vapour pressures and related thermodynamic properties of polycyclic aromatic hydrocarbons determined by gas chromatography, *J.Chromatogr.A*, 2006, 1135 91-100. https://doi.org/10.1016/j.chroma.2006.09.050

13. Chickos, J.S.; Hanshaw, W. Vapor pressures and vaporization enthalpies of the n-alkanes from C21 to C30 at T= 298.15 K by correlation gas chromatography. *J.Chem.Eng.Data* 2004, 49, 77-85.

14. Emmenegger, F.; Piccand, M. Vapour pressure measurements with a thermobalance. *J.ThermAnal.Calorim.* 1999, 57, 235-240. https://doi.org/10.1023/a:1010100531350

15. Chickos, J.S.; Hesse, D.; Hosseini, S.; Nichols, G.; Webb, P. Sublimation enthalpies at 298.15 K using correlation gas chromatography and differential scanning calorimetry measurements. *Thermochim.Acta* 1998, 313, 101-110. https://doi.org/10.1016/S0040-6031(97)00432-2

16. Ruzicka, K.; Majer, V.J. Simultaneous treatment of vapor pressures and related thermal data betweenthe triple and normal boiling temperatures for n-alkanes C5-C20. *J.Phys.Chem.Ref.Data* 1994, 23, 1-39. https://doi.org/10.1063/1.555942

17. Mortimer, F. S.; Murphy, R. V. The Vapor Pressures of Some Substances Found in Coal Tar., *Ind.Eng.Chem.* 1923, 15, 1140. https://doi.org/10.1021/ie50167a012

18. Nelson, O.A.; Senseman, C.E. Vapor Pressure Determinations on Naphthalene, Anthracene, Phenanthrene, and Anthraquinone between Their Melting and Boiling Points. *Ind.Eng.Chem.* 1922. 14, 58-62.

Table S6: Comparison of sublimation enthalpies of pyrene at $\boldsymbol{T}$=298.15 K calculated by two routes[a]

$\Delta_{cr}^{l}H_{m}^{o}(T_{fus}/\mathrm{K})$[b] kJ mol^{-1} T_{fus}=422.4	$\Delta_{cr}^{l}H_{m}^{o}(\boldsymbol{T})$[c] kJ mol^{-1}	$\Delta_{l}^{g}H_{m}^{o}(\boldsymbol{T})$[d] kJ mol^{-1}	$\Delta_{cr}^{l}H_{m}^{o}(\boldsymbol{T})$ $+\Delta_{l}^{g}H_{m}^{o}(\boldsymbol{T})$ kJ mol^{-1}	$\Delta_{cr}^{g}H_{m}^{o}(\boldsymbol{T})$[e] kJ mol^{-1}
16.5 ± 0.5	10.5 ± 0.6	91.4 ± 1.4	101.9 ± 1.5	101.1 ± 5.2

[a] All uncertainties correspond to twice the combined standard. [b] The values were taken from Rojas et al.[i] [c] Fusion enthalpies calculated at 298.15 K using equation 8 and the capacities for liquid and solid pyrene were 290.2 and 234.9 J mol^{-1} K^{-1}, respectively.[i] [d,e] This work [i] Rojas, A.; Orozco, E. Measurement of the enthalpies of vaporization and sublimation of solids aromatic hydrocarbons by differential scanning calorimetry. *Thermochim.Acta*, 2003, 405, 93-107. doi:10.1016/S0040-6031(03)00139-4

Table S7: Experimental values of $C_{p,m}$(cr) of TMAc at different temperatures.[a]

T	$C_{p,m}$	T	$C_{p,m}$	T	$C_{p,m}$
K	J· mol^{-1}· K^{-1}	K	J· mol^{-1}· K^{-1}	K	J· mol^{-1}· K^{-1}
295.15	244.29	334.15	276.02	373.15	292.62
296.15	246.18	335.15	276.65	374.15	293.25
297.15	247.65	336.15	277.91	375.15	293.25
298.15	248.00	337.15	275.70	376.15	293.88
299.15	248.70	338.15	277.81	377.15	293.99
300.15	250.38	339.15	278.12	378.15	294.83
301.15	252.69	340.15	278.96	379.15	295.14
302.15	253.53	341.15	279.49	380.15	295.46
303.15	254.59	342.15	280.54	381.15	295.77
304.15	256.06	343.15	281.17	382.15	295.67
305.15	256.79	344.15	281.48	383.15	296.19
306.15	257.42	345.15	282.01	384.15	296.40
307.15	257.74	346.15	282.85	385.15	296.72
308.15	257.95	347.15	283.06	386.15	296.72
309.15	258.26	348.15	283.90	387.15	297.35
310.15	260.68	349.15	284.64	388.15	298.08
311.15	257.95	350.15	285.16	389.15	298.40
312.15	259.94	351.15	285.37	390.15	299.24
313.15	260.89	352.15	286.00	391.15	299.56
314.15	261.94	353.15	286.11	392.15	299.77
315.15	261.10	354.15	286.74	393.15	299.87
316.15	263.20	355.15	287.47	394.15	300.40
317.15	263.62	356.15	288.00	395.15	300.71

Continued on next page

Table S7 – continued from previous page

T	$C_{p,m}$	T	$C_{p,m}$	T	$C_{p,m}$
K	J· mol^{-1}· K^{-1}	K	J· mol^{-1}· K^{-1}	K	J· mol^{-1}· K^{-1}
318.15	263.94	357.15	287.79	396.15	301.34
319.15	264.88	358.15	288.63	397.15	302.08
320.15	266.04	359.15	289.36	398.15	303.65
321.15	266.56	360.15	289.78	399.15	304.07
322.15	267.51	361.15	289.99	400.15	304.39
323.15	268.35	362.15	290.20	401.15	305.02
324.15	269.29	363.15	290.41	402.15	306.07
325.15	270.14	364.15	290.83	403.15	306.28
326.15	270.56	365.15	291.15	404.15	306.59
327.15	271.29	366.15	291.15	405.15	307.33
328.15	272.45	367.15	291.57	406.15	307.86
329.15	272.87	368.15	291.36	407.15	308.17
330.15	273.29	369.15	291.57	408.15	308.80
331.15	274.02	370.15	291.57	409.15	309.75
332.15	274.65	371.15	291.99	410.15	310.27
333.15	275.28	372.15	292.41	411.15	310.90

[a] $U(C_{p,m})$= 6.33 J· mol^{-1}· K^{-1}, which correspond to twice the combined uncertainty with a 95% confidence level, and include the contributions from the calibration and $u(T)$ = 0.1 K. The experiments were performed at an average pressure of 78.8 kPa. Standard uncertainty for P is $u(P)$= 1 kPa.

Table S8: Thermogravimetric data of TMAc to determine vaporization enthalpy

T	m	$dm/dt \cdot 10^9$	$1/T \cdot 10^3$	ln $(dm/dt \cdot \mathrm{T})$
K	mg	kg· s^{-1}	K^{-1}	
		Series 1		
520.15	7.526	15.000	1.923	-11.761
522.15	7.388	15.690	1.915	-11.712
524.15	7.239	16.510	1.908	-11.658
526.15	7.074	17.480	1.901	-11.597
528.15	6.893	18.590	1.893	-11.531
530.15	6.694	19.820	1.886	-11.463
532.15	6.473	21.140	1.879	-11.395
534.15	6.230	22.540	1.872	-11.327
536.15	5.963	24.120	1.865	-11.256
538.15	5.673	25.720	1.858	-11.188
540.15	5.356	27.440	1.851	-11.119
542.15	5.016	29.190	1.845	-11.054
544.15	4.652	31.020	1.838	-10.989
546.15	4.264	32.950	1.831	-10.925
548.15	3.853	35.000	1.824	-10.861
550.15	3.416	37.040	1.818	-10.801
552.15	2.954	39.020	1.811	-10.745
554.15	2.468	40.980	1.805	-10.693
556.15	1.961	42.740	1.798	-10.647

$\ln(dm/dt \cdot T)$ = 6.15 - 9329.32/T, r^2 = 0.9987, σ_m = 80.32, σ_y = 0.15

$\Delta_l^g H_m^o$(538.15K) = (77.6 ± 0.7)[a] kJ· mol^{-1}

Continued on next page

Table S8 – continued from previous page

T	m	$dm/dt \cdot 10^9$	$1/T \cdot 10^3$	ln $(dm/dt \cdot \mathrm{T})$
K	mg	kg$\cdot$ s^{-1}	K^{-1}	
		Series 2		
520.15	7.749	15.820	1.923	-11.708
522.15	7.603	16.390	1.915	-11.669
524.15	7.446	17.150	1.908	-11.619
526.15	7.274	18.140	1.901	-11.560
528.15	7.085	19.230	1.893	-11.497
530.15	6.877	20.450	1.886	-11.432
532.15	6.649	21.770	1.879	-11.366
534.15	6.399	23.220	1.872	-11.298
536.15	6.124	24.770	1.865	-11.229
538.15	5.825	26.450	1.858	-11.160
540.15	5.499	28.140	1.851	-11.094
542.15	5.151	29.880	1.845	-11.031
544.15	4.780	31.620	1.838	-10.970
546.15	4.386	33.490	1.831	-10.909
548.15	3.968	35.420	1.824	-10.849
550.15	3.526	37.390	1.818	-10.792
552.15	3.062	39.420	1.811	-10.735
554.15	2.571	41.390	1.805	-10.683
556.15	2.060	43.120	1.798	-10.638

$\ln(dm/dt \cdot T)$ = 5.58 - 9012.56/T, r^2 = 0.9983, σ_m = 89.13, σ_y = 0.17

$\Delta_l^g H_m^o$(538.15K) = (74.9 ± 0.7)[a] kJ$\cdot$ mol^{-1}

Continued on next page

Table S8 – continued from previous page

T	m	$dm/dt\cdot 10^9$	$1/T\cdot 10^3$	ln $(dm/dt\cdot \mathrm{T})$
K	mg	kg$\cdot$ s^{-1}	K^{-1}	
		Series 3		
520.15	7.233	15.940	1.923	-11.700
522.15	7.083	16.640	1.915	-11.654
524.15	6.920	17.550	1.908	-11.596
526.15	6.741	18.630	1.901	-11.533
528.15	6.545	19.850	1.893	-11.466
530.15	6.326	21.150	1.886	-11.398
532.15	6.086	22.540	1.879	-11.331
534.15	5.823	24.050	1.872	-11.262
536.15	5.535	25.650	1.865	-11.194
538.15	5.223	27.330	1.858	-11.127
540.15	4.885	29.080	1.851	-11.061
542.15	4.524	30.890	1.845	-10.997
544.15	4.138	32.840	1.838	-10.932
546.15	3.727	34.820	1.831	-10.870
548.15	3.291	36.860	1.824	-10.810
550.15	2.831	38.860	1.818	-10.753
552.15	2.349	40.720	1.811	-10.703
554.15	1.844	42.220	1.805	-10.663
556.15	1.327	43.230	1.798	-10.636
$\ln(dm/dt \cdot T)$ = 5.71 - 9064.07/T, r^2 = 0.9977, σ_m = 105.48, σ_y = 0.20				
$\Delta_l^g H_m^o$(538.15K) = (75.4 ± 0.9)^a kJ$\cdot$ mol^{-1}				

Continued on next page

Table S8 – continued from previous page

T	m	$dm/dt \cdot 10^9$	$1/T \cdot 10^3$	ln $(dm/dt \cdot \mathrm{T})$
K	mg	kg$\cdot$ s^{-1}	K^{-1}	
		Series 4		
520.15	7.552	16.210	1.923	-11.684
522.15	7.402	16.870	1.915	-11.640
524.15	7.238	17.760	1.908	-11.585
526.15	7.059	18.860	1.901	-11.521
528.15	6.861	20.080	1.893	-11.454
530.15	6.642	21.410	1.886	-11.386
532.15	6.400	22.850	1.879	-11.317
534.15	6.135	24.320	1.872	-11.251
536.15	5.845	25.940	1.865	-11.183
538.15	5.530	27.680	1.858	-11.114
540.15	5.189	29.480	1.851	-11.048
542.15	4.824	31.350	1.845	-10.983
544.15	4.432	33.280	1.838	-10.919
546.15	4.017	35.230	1.831	-10.858
548.15	3.575	37.240	1.824	-10.799
550.15	3.111	39.340	1.818	-10.741
552.15	2.620	41.390	1.811	-10.686
554.15	2.105	43.280	1.805	-10.638
556.15	1.571	44.780	1.798	-10.600

$\ln(dm/dt \cdot T) = 5.87 - 9140.82/T$, $r^2 = 0.9985$, $\sigma_m = 85.29$, $\sigma_y = 0.16$

$\Delta_l^g H_m^o(538.15\mathrm{K}) = (76.0 \pm 0.7)^{a}$ kJ$\cdot$ mol^{-1}

Weighted average value $<\Delta_l^g H_m^o(538.15\mathrm{K}) = (76.0 \pm 0.7)>^{b}$ kJ$\cdot$ mol^{-1}

Continued on next page

Table S8 – continued from previous page

T	m	$dm/dt\cdot 10^9$	$1/T\cdot 10^3$	$\ln\,(dm/dt{\cdot}\mathrm{T})$
K	mg	kg$\cdot$ s^{-1}	K^{-1}	
		$<\Delta_l^g H_m^o(298.15\mathrm{K}) = (97.1 \pm 1.4)>^{\mathrm{c}}$ kJ$\cdot$ mol^{-1}		

[a] The uncertainty for each enthalpy of vaporization value was calculated with standard deviation of the slope, which is a standard uncertainty. [b] The weighted average value μ and its standard deviation σ, were calculated as $\mu = \sum(x_i/\sigma_i^2)/\sum(1/\sigma_i^2)$ and σ^2 =N/$\sum(1/\sigma_i^2)$], where x$_i$ is each of the N vaporization enthalpy data and its respective standard deviation σ_i. The experiments of vaporization were realized under average atmospheric pressure (78.8 kPa), u(P)= 1 kPa. The uncertainty corresponds to combined uncertainty. [c] The standard molar enthalpy of vaporization was obtained by equations 11, 12 and 13.

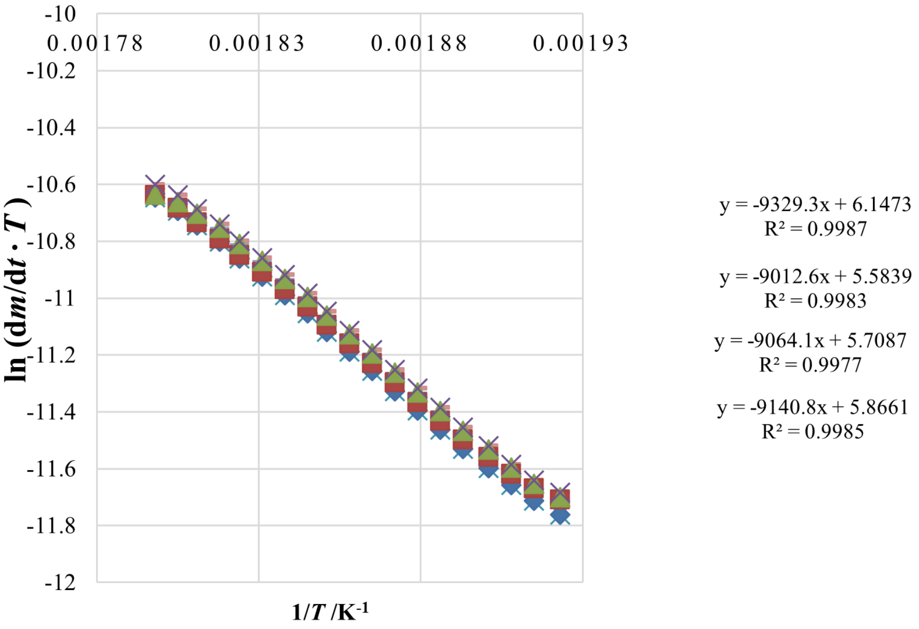

Figure S6: Linear regression of TMAc data as function of $1/T$ to determinate the enthalpy of vaporization, $\Delta_l^g H_m(T_m)$.

Table S9: Thermogravimetric data of TMAc to determine sublimation enthalpy

T	m	$dm/dt\cdot 10^9$	$1/T\cdot 10^3$	ln $(dm/dt\cdot \mathrm{T})$
K	mg	kg· s^{-1}	K^{-1}	
		Series 1		
485.15	10.16	0.574	2.061	-15.094
486.15	10.15	0.622	2.057	-15.012
487.15	10.15	0.658	2.053	-14.953
488.15	10.14	0.697	2.049	-14.894
489.15	10.14	0.741	2.044	-14.830
490.15	10.14	0.776	2.040	-14.782
491.15	10.13	0.809	2.036	-14.738
492.15	10.13	0.879	2.032	-14.653
493.15	10.12	0.935	2.028	-14.590
494.15	10.11	0.983	2.024	-14.538
495.15	10.11	1.061	2.020	-14.459
496.15	10.10	1.134	2.016	-14.391
497.15	10.09	1.248	2.011	-14.293
498.15	10.09	1.358	2.007	-14.206
499.15	10.08	1.481	2.003	-14.118
$\ln(dm/dt\cdot T)$ = 18.17 - 16146.52T, r^2 = 0.9932, σ_m = 370.11, σ_y = 0.75				
$\Delta_l^g H_m^o$(492.15K) = (141.3 ± 4.1)[a] kJ· mol^{-1}				
		Series 2		
485.15	9.476	0.898	2.061	-14.646
486.15	9.471	0.931	2.057	-14.608
487.15	9.465	1.011	2.053	-14.524

Continued on next page

Table S9 – continued from previous page

T	m	$dm/dt\cdot 10^9$	$1/T\cdot 10^3$	ln $(dm/dt\cdot$T)
K	mg	kg$\cdot$ s^{-1}	K^{-1}	
488.15	9.458	1.091	2.049	-14.446
489.15	9.452	1.148	2.044	-14.393
490.15	9.445	1.211	2.040	-14.337
491.15	9.437	1.307	2.036	-14.259
492.15	9.429	1.407	2.032	-14.183
493.15	9.420	1.487	2.028	-14.126
494.15	9.411	1.570	2.024	-14.069
495.15	9.401	1.700	2.020	-13.988
496.15	9.390	1.841	2.016	-13.906
497.15	9.379	1.973	2.011	-13.835
498.15	9.367	2.134	2.007	-13.754
499.15	9.353	2.340	2.003	-13.660
ln$(dm/dt\cdot T)$ = 20.00 - 16820.02/T, r^2 = 0.9972, σ_m = 245.17, σ_y = 0.50				
$\Delta_l^g H_m^o$(492.15K) = (139.8 ± 2.0)[a] kJ$\cdot$ mol^{-1}				
Series 3				
485.15	9.532	0.472	2.061	-15.290
486.15	9.529	0.505	2.057	-15.220
487.15	9.526	0.524	2.053	-15.181
488.15	9.523	0.555	2.049	-15.121
489.15	9.520	0.589	2.044	-15.060
490.15	9.516	0.638	2.040	-14.978
491.15	9.512	0.669	2.036	-14.928
492.15	9.508	0.699	2.032	-14.883

Continued on next page

Table S9 – continued from previous page

T	m	$dm/dt\cdot 10^9$	$1/T\cdot 10^3$	ln $(dm/dt{\cdot}\mathrm{T})$
K	mg	kg$\cdot$ s^{-1}	K^{-1}	
493.15	9.504	0.771	2.028	-14.783
494.15	9.499	0.806	2.024	-14.736
495.15	9.494	0.878	2.020	-14.649
496.15	9.488	0.950	2.016	-14.568
497.15	9.482	1.024	2.011	-14.491
498.15	9.476	1.138	2.007	-14.383
499.15	9.469	1.277	2.003	-14.266

$\ln(dm/dt \cdot T)$ = 18.17 - 16146.62/T, $\mathrm{r}^2 = 0.9932$, $\sigma_m = 370.11$, $\sigma_y = 0.75$

$\Delta_l^g H_m^o(492.15\mathrm{K}) = (134.2 \pm 3.1)^{\mathrm{a}}$ kJ$\cdot$ mol^{-1}

Weighted average value $<\Delta_l^g H_m^o(492.15\mathrm{K}) = (138.6 \pm 2.7)>^{\mathrm{b}}$ kJ$\cdot$ mol^{-1}

$<\Delta_l^g H_m^o(298.15\mathrm{K}) = (144.8 \pm 5.4)>^{\mathrm{c}}$ kJ$\cdot$ mol^{-1}

[a] The uncertainty for each enthalpy of sublimation value was calculated with standard deviation of the slope, which is a standard uncertainty. [b] The weighted average value μ and its standard deviation σ, were calculated as $\mu = \sum(x_i/\sigma_i^2)/\sum(1/\sigma_i^2)$ and σ^2 =N/$\sum(1/\sigma_i^2)$], where x_i is each of the N sublimation enthalpy data and its respective standard deviation σ_i. The experiments of sublimation were realized under average atmospheric pressure (78.8 kPa), u(P)= 1 kPa. The uncertainty corresponds to combined uncertainty. [c] The standard molar enthalpy of sublimation was obtained by equation 15.

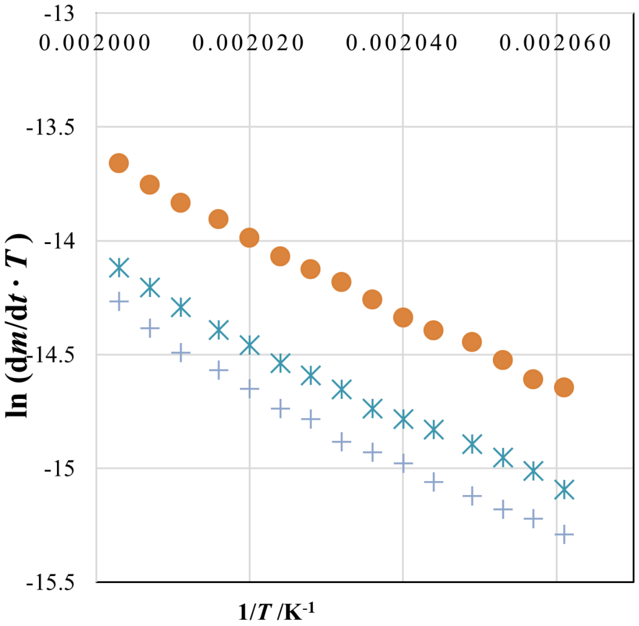

Figure S7: Linear regression of TMAc data as function of $1/T$ to determinate the enthalpy of sublimation, $\Delta_{cr}^{g} H_m(T_m)$.

Table S10 shows an example of how to perform the prediction manually with this method, the correction factors added to this method were the functional group to which the compounds belong, radical 1-4 where it is specified where the substituents are positioned within a molecule (excluding the base compound), i.e., in the case of benzoic acid as there is no difference where the COOH group is positioned that parameter does not consider, If a compound has several corrections due to aromaticity (for example, two meta corrections), correction 1 meta, correction 2 meta, correction 3 and correction 4 will be used, the latter indicating that the compound in question only has 2 corrections for aromaticity instead of 4, which is the maximum that the model can predict, in the case of a compound that does not have corrections for aromaticity, the values of corrections 1-4 will be used (this indicates that the molecule does not have corrections ortho, meta or para within its structure), in addition a starting value H_{f0} must be used, which represents the intersection (a_0) obtained by the regression.

Table S10: Calculation of formation enthalpy in kJ mol^{-1}.

**Trimellitic acid**		
(3*CO-(O)(C_B))+(3*O-(H)(CO))+(3*C_B-(CO)(C_B)$_2$)+(3*C_B-(H)(C_B)$_2$)+(2*radical 1)+ (4*radical 2)+(Correction 1 ortho)+(Correction 2 para)+(Correction 3)+(Correction 4)+ H_{f0}		
$\Delta_f H^\circ_m$(g, 298.15K)	-1034.8	H_{f0}=-243.23 kJ mol^{-1}
$\Delta_f H^\circ_m$(cr, 298.15K)	-1177.5	H_{f0}=-194.18 kJ mol^{-1}
**Trimesic acid**		
(3*CO-(O)(C_B))+(3*O-(H)(CO))+(3*C_B-(CO)(C_B)$_2$)+(3*C_B-(H)(C_B)$_2$)+(3*radical 1)+ (5*radical 2)+(Correction 1 meta)+(Correction 2 meta)+(Correction 3)+(Correction 4)+ H_{f0}		
$\Delta_f H^\circ_m$(g, 298.15K)	-1045.0	H_{f0}=-243.23 kJ mol^{-1}
$\Delta_f H^\circ_m$(cr, 298.15K)	-1189.9	H_{f0}=-194.18 kJ mol^{-1}
**4-methoxybenzoic acid**		
(4*C_B-(H)(C_B)$_2$)+(C_B-(CO)(C_B)$_2$)+(C_B-(O)(C_B)$_2$)+(CO-(O)(C_B))+(O-(H)(CO))+ (O-(C)(C_B))+(C-(H)$_3$(O))+(4*radical 1)+(Correction 1)+(Correction 2)+(Correction 3) +(Correction 4)+ H_{f0}		
$\Delta_f H^\circ_m$(g, 298.15K)	-449.0	H_{f0}=-243.23 kJ mol^{-1}
$\Delta_f H^\circ_m$(cr, 298.15K)	-562.3	H_{f0}=-194.18 kJ mol^{-1}
**Decanoic acid**		
(C-(H)$_3$(C))+(7*C-(H)$_2$(C)$_2$)+(C-(H)$_2$(CO)(C))+(CO-(C)(CO))+(O-(H)(CO))+ (Correction 1)+(Correction 2)+(Correction 3)+(Correction 4)+ H_{f0}		
$\Delta_f H^\circ_m$(g, 298.15K)	-603.9	H_{f0}=-243.23 kJ mol^{-1}
$\Delta_f H^\circ_m$(cr, 298.15K)	-719.4	H_{f0}=-194.18 kJ mol^{-1}

Table S11: Group reparametrization

Predictor	*Description*	*Possible values*
CO-(O)(CO)	A carbonyl group attached to an oxygen and another carbonyl group.	Type: Discrete 0 (absence), 1 to infinity (presence).
CO-(CD)(O)	A carbonyl group attached to a carbon double bond and an oxygen.	Type: Discrete 0 (absence), 1 to infinity (presence).
CO-(C)(O)	A carbonyl group attached to a carbon and an oxygen.	Type: Discrete 0 (absence), 1 to infinity (presence).

Continued on next page

Table S11 – continued from previous page

CO-(H)(O)	A carbonyl group attached to a hydrogen and an oxygen.	Type: Discrete 0 (absence), 1 to infinity (presence).
CO-(O)(CB)	A carbonyl group attached to an oxygen and an aromatic carbon.	Type: Discrete 0 (absence), 1 to infinity (presence).
O-(H)(CO)	An oxygen attached to a hydrogen and a carbonyl group.	Type: Discrete 0 (absence), 1 to infinity (presence).
O-(C)(CB)	An oxygen attached to a carbon and an aromatic carbon.	Type: Discrete 0 (absence), 1 to infinity (presence).
CD-(H)(CO)	A carbon double bond attached to a hydrogen and a carbonyl group.	Type: Discrete 0 (absence), 1 to infinity (presence).
CB-(CO)(CB)2	An aromatic carbon attached to a carbonyl group and two aromatic carbons.	Type: Discrete 0 (absence), 1 to infinity (presence).
CB-(O)(CB)2	An aromatic carbon attached to an oxygen and two aromatic carbons.	Type: Discrete 0 (absence), 1 to infinity (presence).
C-(CO)(C)3	A carbon attached to a carbonyl and three carbons.	Type: Discrete 0 (absence), 1 to infinity (presence).
C-(H)2(CO)(C)	A carbon attached to two hydrogens, a carbonyl, and another carbon.	Type: Discrete 0 (absence), 1 to infinity (presence).
C-(H)3(O)	A carbon atom attached to three hydrogens and an oxygen.	Type: Discrete 0 (absence), 1 to infinity (presence).
C-(H)3(C)	A carbon atom attached to three hydrogens and another carbon.	Type: Discrete 0 (absence), 1 to infinity (presence).
C-(H)3(CB)	A carbon atom attached to three hydrogens and an aromatic carbon.	Type: Discrete 0 (absence), 1 to infinity (presence).

Continued on next page

Table S11 – continued from previous page

C-(H)2(C)2	A carbon atom attached to two hydrogens and two carbons.	Type: Discrete 0 (absence), 1 to infinity (presence).
CD-(H)2	A carbon double bond attached to two oxygens.	Type: Discrete 0 (absence), 1 to infinity (presence).
CB-(H)(CB)2	An aromatic carbon attached to a hydrogen and two aromatic carbons.	Type: Discrete 0 (absence), 1 to infinity (presence).
CB-(C)(CB)2	An aromatic carbon attached to a carbon and two aromatic carbons.	Type: Discrete 0 (absence), 1 to infinity (presence).
C-(H)2(C)(CB)	A carbon atom attached to two hydrogens, a carbon, and an aromatic carbon.	Type: Discrete 0 (absence), 1 to infinity (presence).
CH3(qua)	A methyl group attached to a quaternary carbon.	Type: Discrete 0 (absence), 1 to infinity (presence).
O-(H)(CB)	An oxygen attached to a hydrogen and an aromatic carbon.	Type: Discrete 0 (absence), 1 to infinity (presence).
C-(H)(CO)(C)2	A carbon atom attached to a hydrogen, a carbonyl group, and two other carbon atoms.	Type: Discrete 0 (absence), 1 to infinity (presence).
C-(H)(C)3	A carbon atom attached to a hydrogen and three other carbon atoms.	Type: Discrete 0 (absence), 1 to infinity (presence).
CH3(tert)	A methyl group attached to a tertiary carbon.	Type: Discrete 0 (absence), 1 to infinity (presence).
O-(H)(C)	An oxygen attached to a hydrogen and a carbon.	Type: Discrete 0 (absence), 1 to infinity (presence).

Continued on next page

Table S11 – continued from previous page

C-(H)2(CO)2	A carbon atom attached to two hydrogens and two carbonyl groups.	Type: Discrete 0 (absence), 1 to infinity (presence).
C-(H)(O)(CO)(C)	A carbon atom attached to a hydrogen, an oxygen, a carbonyl, and another carbon.	Type: Discrete 0 (absence), 1 to infinity (presence).
Radical 1	The position of the first radical within the molecule.	Type: Discrete 0 (absence), 1 to infinity (presence).
Radical 2	The position of the second radical within the molecule.	Type: Discrete 0 (absence), 1 to infinity (presence).
Radical 3	The position of the third radical within the molecule.	Type: Discrete 0 (absence), 1 to infinity (presence).
Radical 4	The position of the fourth radical within the molecule.	Type: Discrete 0 (absence), 1 to infinity (presence).
Correction 1	Correction used in case there are no corrections for aromaticity in the molecule.	Type: dichotomous 0 (absence), 1 (presence).
Correction 1 meta	Correction used for having radicals in the meta position.	Type: dichotomous 0 (absence), 1 (presence).
Correction 1 ortho	Correction used for having radicals in the ortho position.	Type: dichotomous 0 (absence), 1 (presence).
Correction 1 para	Correction used for having radicals in the para position.	Type: dichotomous 0 (absence), 1 (presence).
Correction 2	Correction used in case there are no corrections for aromaticity in the molecule.	Type: dichotomous 0 (absence), 1 (presence).

Continued on next page

Table S11 – continued from previous page

Correction 2 meta	Correction used for having radicals in the meta position and one of the ortho, meta, or para corrections at 1.	Type: dichotomous 0 (absence), 1 (presence).
Correction 2 ortho	Correction used for having radicals in the ortho position and one of the ortho, meta, or para corrections at 1.	0 (absence), 1 (presence).
Correction 2 para	Correction used for having radicals in the para position and one of the ortho, meta, or para corrections at 1.	Type: dichotomous 0 (absence), 1 (presence).
Correction 3	Correction used in case there are no corrections for aromaticity in the molecule.	Type: dichotomous 0 (absence), 1 (presence).
Correction 3 meta	Correction used for having radicals in the meta position and one of the ortho, meta, or para corrections at 1 and 2.	Type: dichotomous 0 (absence), 1 (presence).
Correction 3 ortho	Correction used for having radicals in the ortho position and one of the ortho, meta, or para corrections at 1 and 2.	Type: dichotomous 0 (absence), 1 (presence).
Correction 3 para	Correction used for having radicals in the para position and one of the ortho, meta, or para corrections at 1 and 2.	Type: dichotomous 0 (absence), 1 (presence).
Correction 4	Correction used in case there are no corrections for aromaticity in the molecule.	Type: dichotomous 0 (absence), 1 (presence).

Continued on next page

Table S11 – continued from previous page

Correction 4 meta	Correction used for having radicals in the meta position and one of the ortho, meta, or para corrections at 1, 2, and 3.	Type: dichotomous 0 (absence), 1 (presence).
Correction 4 ortho	Correction used for having radicals in the ortho position and one of the ortho, meta, or para corrections at 1, 2, and 3.	Type: dichotomous 0 (absence), 1 (presence).
